# Exercise Prevents Weight Gain and Alters the Gut Microbiota in a Mouse Model of High Fat Diet-Induced Obesity

**DOI:** 10.1371/journal.pone.0092193

**Published:** 2014-03-26

**Authors:** Christian C. Evans, Kathy J. LePard, Jeff W. Kwak, Mary C. Stancukas, Samantha Laskowski, Joseph Dougherty, Laura Moulton, Adam Glawe, Yunwei Wang, Vanessa Leone, Dionysios A. Antonopoulos, Dan Smith, Eugene B. Chang, Mae J. Ciancio

**Affiliations:** 1 Department of Physical Therapy, College of Health Sciences, Midwestern University, Downers Grove, Illinois, United States of America; 2 Department of Physiology, College of Osteopathic Medicine, Midwestern University, Downers Grove, Illinois, United States of America; 3 Department of Biomedical Sciences, College of Health Sciences, Midwestern University, Downers Grove, Illinois, United States of America; 4 College of Osteopathic Medicine, Midwestern University, Downers Grove, Illinois, United States of America; 5 Department of Dental Medicine, College of Dental Medicine, Midwestern University, Downers Grove, Illinois, United States of America; 6 Department of Medicine, University of Chicago, Chicago, Illinois, United States of America; 7 Institute for Genomics and Systems Biology, Argonne National Laboratory, Argonne, Illinois, United States of America; University of Tor Vergata, Italy

## Abstract

**Background:**

Diet-induced obesity (DIO) is a significant health concern which has been linked to structural and functional changes in the gut microbiota. Exercise (Ex) is effective in preventing obesity, but whether Ex alters the gut microbiota during development with high fat (HF) feeding is unknown.

**Objective:**

Determine the effects of voluntary Ex on the gastrointestinal microbiota in LF-fed mice and in HF-DIO.

**Methods:**

Male C57BL/6 littermates (5 weeks) were distributed equally into 4 groups: low fat (LF) sedentary (Sed) LF/Sed, LF/Ex, HF/Sed and HF/Ex. Mice were individually housed and LF/Ex and HF/Ex cages were equipped with a wheel and odometer to record Ex. Fecal samples were collected at baseline, 6 weeks and 12 weeks and used for bacterial DNA isolation. DNA was subjected both to quantitative PCR using primers specific to the 16S rRNA encoding genes for Bacteroidetes and Firmicutes and to sequencing for lower taxonomic identification using the Illumina MiSeq platform. Data were analyzed using a one or two-way ANOVA or Pearson correlation.

**Results:**

HF diet resulted in significantly greater body weight and adiposity as well as decreased glucose tolerance that were prevented by voluntary Ex (p<0.05). Visualization of Unifrac distance data with principal coordinates analysis indicated clustering by both diet and Ex at week 12. Sequencing demonstrated Ex-induced changes in the percentage of major bacterial phyla at 12 weeks. A correlation between total Ex distance and the ΔCt Bacteroidetes: ΔCt Firmicutes ratio from qPCR demonstrated a significant inverse correlation (r^2^ = 0.35, p = 0.043).

**Conclusion:**

Ex induces a unique shift in the gut microbiota that is different from dietary effects. Microbiota changes may play a role in Ex prevention of HF-DIO.

## Introduction

Approximately 35% of adults [1] and 17% of adolescents and children in the United States are obese [2] and this epidemic is fueling a rise in type-2 diabetes, metabolic syndrome and heart disease [3]. The financial burden on an already stressed economy has been estimated to be $147 billion/year in medical care alone in the US (2008 estimate) [4]. Lifestyle changes or other treatments that can prevent or limit obesity would significantly impact the health of the nation as well as reduce the socioeconomic burden on society. On average, Americans consume approximately 34% of their total dietary calories from fat [5], more than recommended by the American Heart Association [6]. High fat (HF) diets are known to induce metabolic stress on the body, leading to obesity and low grade systemic inflammation [7].

Recent research indicates that the gut microbiota induces the development of obesity in both humans [8] and mouse models [9], [10]. Studies have shown that obese individuals have an imbalance in the primary bacterial phyla comprising the gastrointestinal (GI) microbiota: Bacteroidetes and Firmicutes [8], [10], [11]. Phyla level shifts are reported with HF, Western diets that appear to directly impact host metabolism and contribute to the development of obesity [10], [12]. In addition, sub-phylum level changes with a HF diet have also been reported, including an increase in the Mollicutes [12] and Erysipelotrichi classes [13].

While the relationship between diet-induced changes in gut microbiota may be model dependent, “microbiota transplant” models using germ-free mice demonstrated that gut microbes from obese mice have a direct effect in increasing fat mass and body weight compared to introduction of microbes from lean mice [10], [12]. Moreover, the obese-associated gut microbiota have increased capacity for energy harvest from ingested food [10] and when weight gain is controlled in the design, it is the HF diet, not body fatness, that determines whether a gut microbiota shift occurs [14].

Exercise (Ex) is known to confer numerous health-related benefits. Recent studies indicate that Ex can normalize body weight, body fat, hyperglycemia and markers of inflammation in mouse models of diet-induced obesity (DIO) [15], [16]. In fact, Ex has been reported to share many of the same anti-inflammatory effects as calorie restriction in terms of reversing obesity and insulin resistance in a model of HF-DIO [16]. Exercise also reduces the incidence of a number of GI-related disorders such as colon cancer [17], [18] and diverticular disease [19]. A study by Oliveira et al. suggested that physical Ex attenuates the inflammatory response of HF feeding [20], but whether this effect is mediated by activity-induced changes in the gut microbiota was not explored.

Few studies have specifically examined the effect of Ex on the gut microbiota. An early study by Matsumoto et al. examined the effect of chronic, load-bearing Ex on cecal microbiota in a non-obese rat model [21]. In the Matsumoto study, forced running altered cecal *n*-butyrate concentration as a consequence of a corresponding change in the *n*-butyrate producing bacteria compared to sedentary rats. More recently, Choi et al. examined the effect of Ex on polychlorinated biphenyl metabolism and the gut microbiota. They found that Ex altered the gut microbiota in normal chow-fed mice, predominantly affecting the families *Erysipelotrichaceae* and *Enterococcaceae* [22]. Whether Ex directly alters the gut microbiota, preventing HF diet-induced changes during development is not known.

In this study, we tested the hypothesis that voluntary Ex would reshape the gut microbiota in both LF and HF-fed mice and that Ex would normalize the gut microbiota in HF mice at the phylum level, in addition to mitigating the development of HF-DIO. Our results indicate that HF diets do indeed promote increased fatness, hyperglycemia and an altered gut microbiota profile compared to LF fed mice. Voluntary Ex prevented these changes in HF mice and resulted in restoration of the phylotypic profile observed with LF feeding. There was a significant correlation between the distance run and alterations in the major gut bacterial phyla for the Ex mice. In addition, differences were also observed at the sub-phylum level between the LF and HF groups with Ex. These findings may offer new insights into the beneficial effect of regular Ex in preventing DIO by modifying the bacterial balance of the gut microbiota.

## Methods

### Animals

All experimental protocols were approved by the Midwestern University Institutional Animal Care and Use Committee (IACUC) and all procedures were performed in accordance with Midwestern University's guidelines for the care and use of laboratory animals. Five week old, male C57BL/6 mice (n = 48) (Jackson Laboratories, Bar Harbor, ME, USA) were received as littermates (15–19 g) and allowed to acclimate in Midwestern University's animal facility for 1 week prior to their random assignment to one of 4 diet and activity treatment groups. During the acclimation period, mice were kept in their respective litters and provided standard chow (composition: 18 kcal% fat, 58 kcal% carbohydrate, 24 kcal% protein; 3.1 kcal/g total energy content, Teklad Diets, Madison WI) and reverse osmosis water *ad libitum*.

### Diet and activity protocol

Following the acclimation phase, mice were randomly assigned to diet and activity groups based on equal distribution of littermates (i.e. mice in each litter were distributed as equally as possible among the 4 groups). Dietary intake included either high fat diet (HF; composition 60 kcal% fat, 20 kcal% carbohydrates, 20 kcal% protein; 5.24 kcal/g total energy content) or low fat diet (LF; composition 10 kcal% fat, 70 kcal% carbohydrates, 20 kcal% protein; 3.85 kcal/g of food, Research Diets, Inc.; New Brunswick, NJ). Mice were individually housed in rat cages in the absence (sedentary; Sed) or presence (exercise; Ex) of a non-load bearing 14.6 cm hamster wheel (PetSmart, Phoenix, AZ) suspended from a metal rod based on a design reported by Konhilas et al. [23]. The cages were modified to record wheel movement 24 h/day, 7 days/week, using Schwinn 17-function odometers (Schwinn, Madison, WI.). The odometers were hand-tested for reliability of time of movement and distance covered. Exercise was recorded continuously (kilometers) and the cages were visually checked at least 3 times/week to ensure the odometers were working correctly and the wheels were functioning properly. Mice were observed exercising during the 12 hour light cycle, but were not disturbed during the dark cycle.

Littermates were separated into groups as follows: LF/Sed, n = 12, LF/Ex, n = 10, HF/Sed, n = 14, and HF/Ex, n = 12. Two separate batches of mice were used: an initial batch of mice were studied for preliminary indications of a global microbiota shift (terminal-restriction fragment length polymorphism (T-RFLP)) and measurement of gut transit (N = 24: LF/Sed, n = 6, LF/Ex, n = 4, HF/Sed, n = 8, and HF/Ex, n = 6) and a second batch for quantitative PCR (qPCR) and sequencing studies (N = 24; n = 6 all groups). All mice were followed on the protocol for 12 weeks for collection of fecal samples and then an additional two weeks for collection of blood samples and harvesting of tissues.

### Weight and food intake measurements

Initial, weekly and final body weights were determined for all mice. Twenty-four hour food intake was measured at week 12 of the experimental period. For these measurements, mice were placed in cages without bedding and each mouse was given one pre-weighed pellet of their respective diet, either LF or HF food, in a ceramic dish designed to minimize food loss or contamination by feces. After 24 hours, the pellet and its remnants were weighed to determine food consumption. Food intake was expressed as kcal/24 hours.

### Blood glucose measurements

Fasting blood glucose (FBG) and oral glucose tolerance (OGT) were measured at 13 weeks on the diet and activity program. Mice were fasted overnight in cages without bedding prior to measuring tail vein blood glucose levels using a OneTouch UltraMini® monitor (LifeScan, Inc. Milpitas, CA). Oral glucose tolerance was measured immediately following the FBG test. Mice were gavaged with 25% glucose in saline (1 g/kg body weight) followed by blood glucose measurements at 15, 30, 60 and 120 minutes post glucose challenge [24] using the tail vein with a lancet stick according to the method of Golde et al. [25].

### Tissue harvest and measurements

At the end of week 13 of the protocol, mice were anesthetized with CO_2_ and then subjected to pneumothorax. Heart and soleus muscles were removed, rinsed several times, and blotted dry to determine their respective wet weights. Epididymal fat pads were excised, rinsed, blotted dry, weighed, and used as indicators of adipose tissue accumulation.

### Intestinal transit

Measurement of intestinal transit was performed as a terminal experiment. Mice were fasted for 16 h with water available *ad libitum*. At 30 minutes prior to euthanasia, 0.1 ml of 0.5 mmol/L 70 kDa fluorescein isothiocyanate (FITC)–dextran (Sigma-Aldrich, St. Louis, MO) was administered by oral gavage [26], [27]. After 30 minutes, mice were euthanized and the entire gastrointestinal tract, from stomach to anus, was removed. The small intestines were cut into 8 segments of equal length from the stomach to the cecum. The stomach and each segment were flushed with 3 ml of 50 mmol/l Tris buffered saline, the effluent was collected and centrifuged at 1200 rpm for 5 minutes to pellet any suspended debris. Fluorescent intensity in each sample was measured using a fluorescence spectrophotometer (emission wavelength  = 518 nm; excitation wavelength  = 492 nm; F-2000, Hitachi, Inc., Japan). Intestinal geometric center (IGC) was calculated as: (fraction of amount of FITC in each segment) × (segment number). The dextran concentration in each segment was expressed as a fraction of total dextran recovery.

### Fecal pellet collection and DNA extraction

Extraction of DNA from fecal bacteria followed the protocol of Wang et al. [28]. Fecal pellets were obtained from all mice at the start (prior to beginning the protocol, week 0), mid-point (week 6), and at week 12 of the diet and Ex protocol. Fecal pellets were collected directly from the mice and stored at −80°C prior to lysis in 1 ml extraction buffer [50 mM Tris (pH 7.4), 100 mM EDTA (pH 8.0), 400 mM NaCl, 0.5% SDS] containing 20 μl proteinase K (20 mg/ml). Bacterial disruption was achieved using 0.1-mm-diameter zirconia/silica beads (BioSpec Products, Bartlesville, OK) and a Mini-Beadbeater-8 k Cell Disrupter (BioSpec Products). The homogenized samples were incubated overnight at 55°C with shaking. The beads were then spun down by centrifugation and 200 μl of room temperature saturated NaCl (175.3 g/500 ml) was added to 500 μl of the supernatant. After 2 minutes of vigorous shaking, the samples were spun for 15 minutes to pellet the protein and debris. Five hundred μl of phenol:chloroform:isoamylalcohol (25∶24∶1;4°C; Ambion) was added to 500 μl of the supernatant. Following shaking and centrifugation, 500 μl of the top phase was mixed with 500 μl of 100% ethanol (EtOH) and placed in −20°C for 30 minutes to precipitate all DNA. Tubes were spun at 4°C for 5 minutes to pellet the DNA, the pellet was washed with 70% EtOH, allowed to air dry at room temperature, and the DNA was dissolved in 50–100 μl of water and stored at −80°C. DNA concentrations and purity were then assessed by spectrophotometry using the NanoDrop® (NanoDrop ND-2000 spectrophotometer; Thermo Scientific, Wilmington, DE). A minimum ratio of 1.9–2.0 was accepted for the 260∶280 ratio.

### Terminal Restriction Fragment Length Polymorphism

The T-RFLP analysis was performed as previously described [28]. Briefly, the 16S rRNA encoding gene was amplified from fecal sample DNA using a broad-range universal primer set: 8F (5′-AGAGTT TGATCCTGGCTCAG-3′) with a 6′ carboxyfluorescein (6-FAM) label and 1492R (5′-GGTTACCTTGTTACGACTT-3′). Each 50 μl PCR reaction contained 50 ng of DNA, 5.0 μl 10× Taq Buffer, 4.0 μl dNTP mixture (2.5 mM each), 0.25 μl TaKaRa Ex Taq (High fidelity Taq Enzyme, 5 units/μl; TaKaRa Mirus Bio, Madison, WI, USA), 1 μl forward primer and 1 μl reverse primer (10 pmol/μl each). PCR was run for 30 cycles using the thermal profiles of: 1 min at 94°C; 30 cycles of (94°C for 30 sec, 58°C for 1 min, 72°C for 1.5 minutes); and a final extension period of 6 min at 72°C. PCR products were verified by electrophoresis and visualization of a band at ∼1400 bp. Upon confirmation, the remaining PCR products were purified and stored at −80°C.

Purified 16S rDNA was digested using 20 U *Msp*I enzyme (New England Biolabs Inc.) in a total volume of 15 μl (including 1.5 μl NE Buffer 2, 1.5 μl enzyme, and 12 μl PCR product) at 37°C for 3 hours. To enhance the fluorescent signal, each digested T-RFLP sample was desalted by single drop analysis. Digested samples were desalted, mixed with deionized formamide and 0.5 μl GeneScan-500 size standard (Applied Biosystems) followed by capillary electrophoresis (Applied Biosystems DNA Sequencer 3130, Life Technologies, Grand Island, NY). Fragment length and abundance were determined using GeneMapper software (Applied Biosystems, Life Technologies, Grand Island, NY). Raw electropherograms were collected and analyzed for artifacts. To visualize relationships between the samples, sample distance values were analyzed by T-RFLP Analysis EXpedited (T-REX) and plotted using principal coordinates analysis (PCA) [29].

### Quantitative PCR

The two gut bacterial phyla of interest, Bacteroidetes and Firmicutes, were targeted by modifying a quantitative PCR assay developed by Armougom et al. using primer and probe sets that recognized a conserved region of the 16S rRNA encoding gene unique to these taxonomic groups [30]. This assay was tested by Armougom et al. on a variety of Bacteroidetes and Firmicutes genera and found to have high sensitivity (>90% for all but one Bacteroidetes subgroup and >80% for all but one Firmicutes subgroup) and a low rate of false positives (0.01% for Bacteroidetes and 0.83% for Firmicutes genera). The efficiency of the assays was determined in the original publication [30]. A broad-range universal primer and probe set that recognize the conserved region of the 16S rRNA encoding gene for a wide variety of bacteria was used to normalize the assay to total bacterial DNA [31]. This primer-probe set was found to have high sensitivity to a broad range of lower taxonomic bacterial groups including members of both the Bacteroidetes and Firmicutes phyla by Nadkarni et al. [31].

The assay was performed on the ABI-PRISM 7700 Sequence Detection System (Applied Biosystems, Life Technologies, Grand Island, NY). The methods on which this assay was based have been published previously [30], [31], but briefly the conditions for quantitative amplification for all qPCR runs were as follows: pre-amplification stage 95°C for 10 min and (40 cycles at 95°C×15 seconds, 60°C×1 minute) for each cycle. The reaction was performed in a 96 well optical grade plate (LifeTechnologies, Inc.) in a total volume of 25 μL using Environmental PCR Master Mix (TaqMan®, LifeTechnologies). Wells contained 400 nM of the probe and 200 nM of the forward and reverse primers for each of the 3 assays and all runs were performed in triplicate (see Table S1 for primer and probe sequences). The amplicon size was 184 base pairs for Bacteroidetes, 179 for Firmicutes, and 466 for the universal set (see Figure S1 for gel showing PCR product size). Prior to qPCR, amplicon size for each target was verified by standard PCR followed by gel electrophoresis and visualization of bands consistent with the appropriate size (see Figure S1). The raw fluorescent signal was analyzed on Sequence Detection Software version 1.4 (Applied Biosystems, LifeTechnologies) and reported as Ct value. The universal Ct value for each respective sample was subtracted from the Ct values for Bacteroidetes and Firmicutes to calculate a ΔCt value for each sample, respectively.

### Sequencing

Microbial sequencing was performed on the MiSeq Illumina platform (Argonne National Laboratory, Institute for Genomics and Systems Biology, Next Generation Sequencing Core) as previously described by Caporaso et al. [32], [33]. Briefly, the V4 region of 16S bacterial rDNA was amplified using the custom degenerate primer pair 515F (GTGCCAGCMGCCGCGGTAA) and 806R (GGACTACHVGGGTWTCTAAT) to generate an amplicon size of 253 base pairs. Reverse primers included a 12 base pair nucleotide barcode to facilitate multiplex sequencing of all 72 samples (detailed in File S1) on a single 150 bp×2 paired-end MiSeq run. The resulting 5,179,000 paired reads were joined using EA-Util's fastq-join script with default parameters, then screened to exclude sequences which contained one or more base calls with a Phred score less than 20.

### Bioinformatics

Bioinformatic analysis of sequencing data was conducted using the QIIME 1.5.0 software suite [33]. Reads from all samples were clustered at 97% sequence identity into operational taxonomic units (OTUs) then aligned to the October 12th, 2012 Greengenes bacterial reference tree [34]. Following removal of OTUs composed of only one sequence, sequence sets for each of the 72 samples were rarefied to an equal sampling depth of 1,600 reads per sample. Unifrac distances between samples were calculated using both weighted and unweighted algorithms and visualized with PCA plots and dendrograms generated with Archeopteryx [35]. Preliminary screening of data for treatment effects was calculated using ANOVA, G Test, ADONIS, ANOSIM, MRPP, PermDISP, RDA, and Student's T-Test algorithms. Commands used in the analysis of this dataset are available in the (File S1). Treatment effects relative to the primary outcome were calculated for specific weeks and between weeks with a two-way and a repeated measure ANOVA, respectively.

### Availability of Supporting Data

The data are publicly available via NCBI GenBank (accession numbers KJ210875 - KJ361456; HF/Ex [KJ210875 - KJ251271], HF/Sed [KJ251272 - KJ288303], LF/Ex [KJ288304 - KJ333958], and LF/Sed [KJ333959 - KJ361456 ]. Additionally, the data is available via a project specific page in MG-RAST (http://metagenomics.anl.gov/linkin.cgi?project=7038) based at Argonne National Laboratory, including instant availability of the sequence data, bioinformatic analyses and tools.

### Statistical Analysis

Quantitative data is represented as the mean ± SEM, unless otherwise indicated. Differences between treatment groups were compared using an independent t-test (for running distance), a Pearson's r for correlation, a one-way ANOVA for qPCR data (on the ΔCt values) and a two-way ANOVA for all other data comparisons on Prism 5 (GraphPad, Inc.) and SPSS 18 (IBM, Inc.) software. Two-way ANOVA results were reported as the F-statistics and p values and a Sidak post-hoc test was used for within group comparisons. To determine the significance of microbial community partitioning due to experimental treatments, permutational multivariate analysis of variance (PERMANOVA) was employed to generate p-values based on the observed Unifrac distance matrix relative to 10,000 randomly rearranged distance matrices. For display purposes only, the effect of exercise on the qPCR fold change in Bacteroidetes and Firmicutes in LF/Ex and HF/Ex mice is shown by the 2^−ΔΔCt^ with the LF/Sed and HF/Sed as the respective comparison groups. A value of p<0.05 was considered statistically significant and p<0.10 was considered to indicate a trend.

## Results

### Characterization of Mouse Model of Voluntary Exercise and Diet-induced Obesity

#### Voluntary Exercise

**Figure 1:**
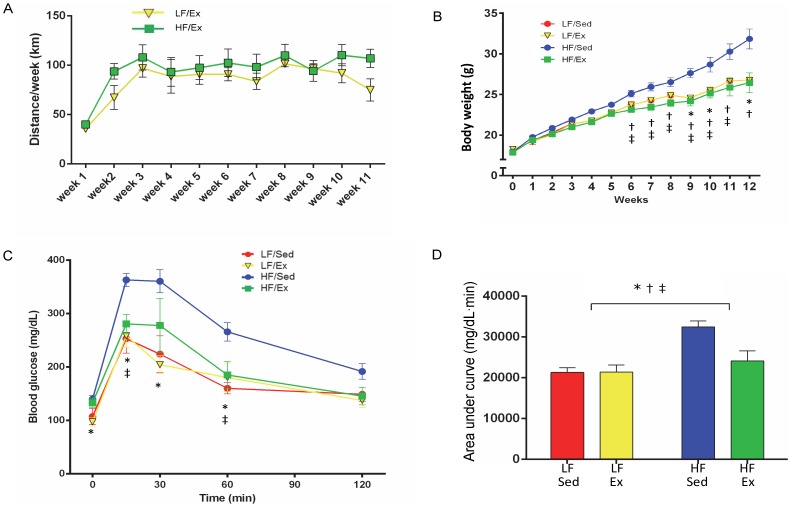
Exercise, Body Weight and Glucose Tolerance. A. Weekly exercise was recorded for low fat/exercise (LF/Ex) and high fat/exercise (HF/Ex) mice. B. Weekly body weight starting at week 0 of the diet and activity protocol (age  = 5 weeks) through week 12 (age  = 17 weeks) was recorded. C. Oral glucose tolerance was tested at week 12 of the diet and activity protocol. D. The area under the curve (AUC) was calculated for oral glucose tolerance. Data are presented as mean ± SEM. Groups are abbreviated as follows: low-fat/sedentary (LF/Sed), low-fat/exercise (LF/Ex), high-fat/sedentary (HF/Sed) and high-fat/exercise (HF/Ex). Data were analyzed by 2-way ANOVA with a Sidak post hoc test. Significant differences indicated as follows: “*” p<0.05 for diet effect, “†” p<0.05 activity effect and “‡” p<0.05 diet and activity interaction. n = 6 mice/group.

Mice in the Ex groups (LF/Ex and HF/Ex) spontaneously ran on the cage-mounted wheel and averaged 82±3.9 km/week and 89±7.9 km/week for the LF/Ex and HF/Ex groups, respectively (p>0.05; Figure 1A). By the end of the protocol, mice accumulated an average total of 985±46.5 km and 1076±95.5 km for the LF/Ex and HF/Ex groups, respectively. Bilateral soleus muscle:body weight ratios and heart:body weight ratios were determined for all groups as indicators of an Ex training effect. Soleus muscle:body weight ratios were increased by Ex (F(1,20) = 17.1, p<0.001), but not diet (F(1,20) = 0.90, p = 0.35), and Ex affected both LF and HF mice similarly (interaction F(1,20) = 0.34, p = 0.57; Table 1). Heart:body weight ratios were increased by Ex (activity F(1,20) = 22.69, p = 0.001) and reduced by HF diet (diet F(1,20) = 6.44, p = 0.02), but there was no interaction (F(1,20) = 0.69, p = 0.41; Table 1).

**Table 1:** Body and fat weight, exercise distance, food intake, heart and muscle weight and gut transit.

	Low fat/sedentary	Low fat/exercise	High fat/sedentary	High fat/exercise	
	N = 6	N = 6(5)	N = 6	N = 6	
Initial body weight (g)	18.1±0.4	18.3±0.3	17.9±0.4	17.9±0.3	
Final body weight (g)	27.1±0.5	25.3±0.2	33.1±1.2	27.9±1.3	*^†^
Total distance run (km)	N/A	985.2±46.5	N/A	1076±95.5	
Epididymal fat pad weight (g)	0.67±0.07	0.47±0.04	1.73±0.27	0.85±0.22	*^†^
24 hour food intake (kcal)	14.2±0.7	17.2±0.7	15.3±0.7	16.0±0.7	^†^
Soleus muscle:body weight (mg/g)	0.84±0.03	1.04±0.02	0.82±0.04	0.97±0.06	^†^
Heart:body weight (mg/g)	4.67±0.29	5.62±0.23	3.85±0.15	5.21±0.27	*^†^
Gut transit (geometric center)	4.69±0.53	4.07±0.58	4.51±0.31	4.22±0.62	

Data represent mean ± SEM. For the low fat/exercise group, one mouse died prior to harvesting tissues. Statistically significant diet effect indicated by * and activity effect by ^†^ based on a two-way ANOVA with a Sidak post hoc test.

#### Diet-induced Obesity

Figure 1B demonstrates the weekly body weight for each group. Mice were 5 weeks old at the start of the diet and Ex protocol (Time 0; Figure 1B), with comparable starting body weights (Table 1). By week 6 of the protocol, there was a significant Ex effect on weight gain (activity F(1,20) = 6.50, p = 0.019) and an interaction (F(1,20) = 6.49, p = 0.019) with HF/Sed mice heavier than HF/Ex mice, but no significant diet effect (F(1,20) = 0.96, p = 0.34). Ex reduced weight gain in the HF mice at all time-points from week 6 through week 12 (p<0.05). By week 9, the HF/Sed mice were heavier than the mice in the other treatment groups (diet F(1,20) = 9.37, p = 0.006; activity F(1,20) = 16.13, p<0.001, interaction F(1,20) = 16.13, p<0.001). The diet effect continued to be significant at weeks 10 and 12. At week 12, there was an effect of both diet (F(1,20) = 21.34, p<0.001) and activity (F(1,20) = 14.21, p = 0.001) on body weight (Table 1, Figure 1B). Consumption of a LF diet and increased physical activity both limited weight gain and showed a tendency for an interaction (F(1,20) = 3.43, p = 0.08).

Metabolically, the HF/Sed mice demonstrated elevated body fat and altered glucose metabolism. Body fat, as measured by epididymal fat pad weight (Table 1), was elevated by HF diet (F(1,20) = 8.07, p = 0.011) and reduced by Ex (F(1,20) = 14.2, p = 0.001), but Ex reduced body fat in both groups with a tendency for an interaction effect (F(1,20) = 3.19, p = 0.09). Oral glucose challenge demonstrated reduced glucose clearance in the HF/Sed mice (Figure 1C). As demonstrated by the area under the curve (Figure 1D), glucose tolerance was impaired by HF diet (F(1,20) = 15.3, p = 0.001) and normalized by Ex (F(1,20) = 5.38, p = 0.031), but Ex only affected glucose tolerance in the HF group (HF/Sed vs. HF/Ex, p = 0.016; interaction F(1,20) = 5.68, p = 0.027).

Twenty-four hour food intake, measured during week 13 of the protocol, was elevated by Ex (F(1,20) = 5.31, p = 0.032), but not diet (F(1,20) = 0.008, p = 0.93), with Ex influencing food intake of LF and HF mice similarly (interaction F(1,20) = 2.17, p = 0.15; Table 1).

Diet and Ex did not significantly influence gastrointestinal transit under fasting conditions. Based on the geometric center measurements reported in Table 1, gut transit time was not affected by either diet (F(1,20)  = 0.001, p = 0.98) or Ex (F(1,20) = 0.735, p = 0.39).

### Diet and Ex-induced Changes in Fecal Bacterial Content

Characterization of changes in the gut bacterial community as a consequence of diet and Ex was examined in three ways using bacterial 16S rRNA encoding genes present in the fecal pellets: terminal restriction fragment length polymorphism (T-RFLP), sequencing, and quantitative PCR (qPCR).

#### Terminal Restriction Fragment Length Polymorphism

T-RFLP was used to determine whether there was a global shift in the gut microbial community after the 12 week diet and activity protocol using the 16S rRNA encoding genes present in the fecal pellets. Subjective analysis of raw electropherograms from T-RFLP analysis demonstrated visible changes in the abundance and distribution of community groups as a result of diet and activity (Figure S2A). Principal coordinates analysis indicated some degree of clustering by litter at the start of the study (Figure S2B), with obvious clustering by diet and activity by week 12 (Figure S2C).

#### Bacterial 16S rRNA Amplicon Sequencing

**Figure 2:**
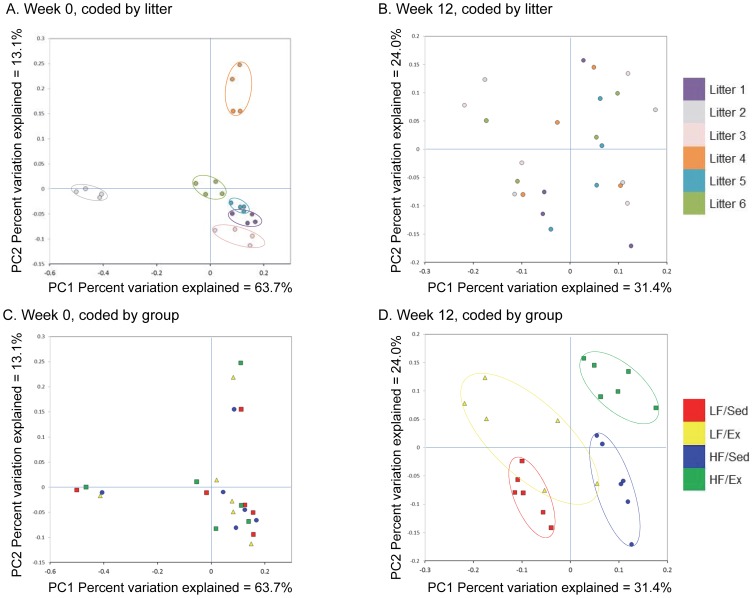
Clustering of Samples Based on Litter, Diet and Activity. Principal coordinate analysis (PCA) was performed based on the weighted UniFrac distance matrix generated from sequencing fecal 16S rRNA gene in samples from mice at week 0 and 12 of the diet and activity protocol. A. Clustering demonstrated by litter at week 0. B. No clustering demonstrated by litter at week 12. C. No clustering demonstrated by diet and activity at week 0. D. Clustering demonstrated by diet and activity at week 12. The top panels show the PCA keyed by litter (6 liters, 1–6, of 4 mice each) and the bottom panels show the PCA keyed by diet and activity group. The X-axis represents the primary coordinate, the Y-axis represents the secondary coordinate. Axis numbering represents the relative distance between samples based on the weighted UniFrac distance matrix.

**Figure 3:**
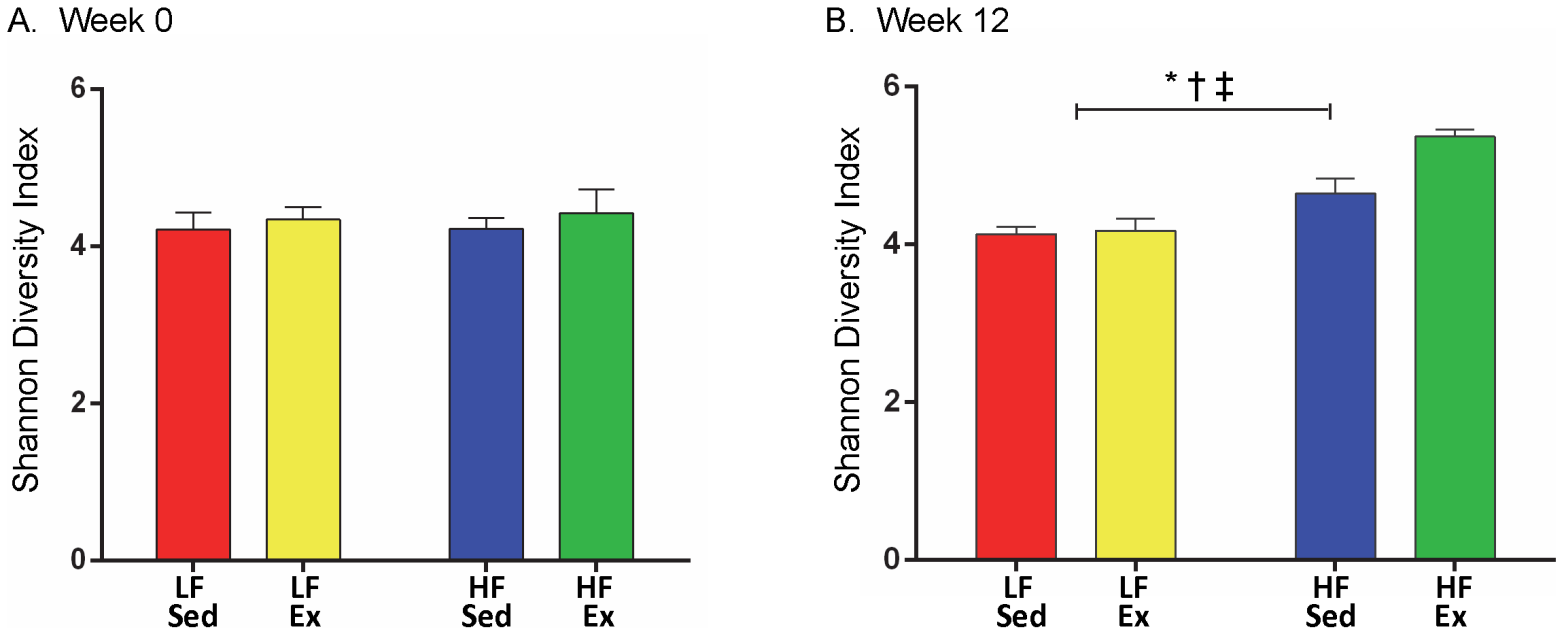
Beta Diversity is Elevated by HF Diet and Exercise. Significant differences indicated as follows: “*” p<0.05 for diet effect, “†” p<0.05 activity effect and “‡” p<0.05 diet and activity interaction. n = 6 mice/group.

Closer analysis of bacterial differences induced by diet and Ex were determined by sequencing the 16S rRNA encoding genes present in the fecal pellets. Principal coordinates analysis of Illumina MiSeq amplicon data demonstrated significant clustering at week 0 based on litter (Figure 2A), which was no longer evident by week 12 (Figure 2B). In contrast, there was no clustering of samples by diet and activity in week 0 (Figure 2C), but robust clustering based on treatment group by week 12 (Figure 2D). PERMANOVA analysis indicated a significant litter effect at week 0 (litter pseudo-F = 4.13, p<0.001), but no diet/activity effect (diet/activity pseudo-F = 0.53, p = 1.0) and only a trend toward a litter effect at week 12 (litter pseudo-F = 1.18, p = 0.064), but a significant diet/activity effect (diet/activity pseudo-F = 2.17, p<0.001). Dendrogram analysis (Figure S3) corroborated the PCA findings, demonstrating clustering by intervention after 6 and 12 weeks on the diet and Ex protocol rather than by litter. The effect of Ex appears to be more distinct in the HF group based on a greater degree of separation of the HF/Ex from the HF/Sed group. The Shannon diversity index (beta diversity) for the samples at the start of the protocol were similar (Figure 3A), but at week 12, both diet (F(1,20) = 39.22, p<0.001) and activity (F(1,20) = 7.8, p = 0.011) increased diversity of the community (Figure 3B), with Ex exerting a greater effect in the HF group (interaction F(1,20 = 6.12, p = 0.022; p<0.001).

**Figure 4:**
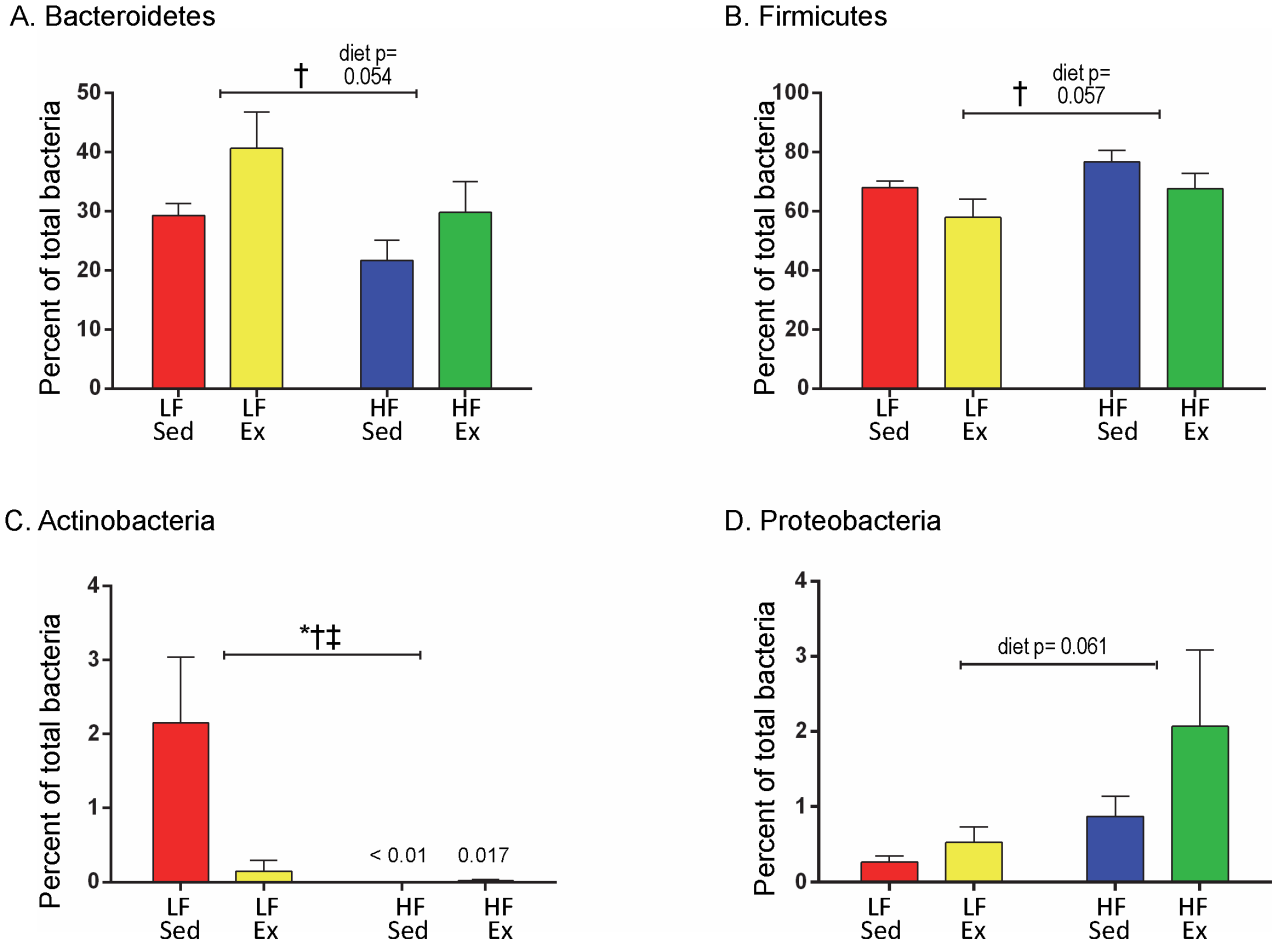
Phylum Level Changes with Diet and Activity. At week 12, diet and activity changed the levels of two major and two minor phyla of bacteria. Data were analyzed by 2-way ANOVA with a Sidak post hoc test. Significant differences indicated as follows: “*” p<0.05 for diet effect, “†” p<0.05 activity effect and “‡” p<0.05 diet and activity interaction. n = 6 mice/group.

Six different bacterial phyla were identified in the MiSeq 16S rRNA based amplicon libraries at the start and completion of the diet and Ex protocol (Bacteroidetes, Firmicutes, Actinobacteria, Proteobacteria, Verrucomicrobia and Tenericutes). While there were no differences at baseline (data not shown), there were measureable differences in the percentage of these bacterial phyla among the 4 diet and activity groups by week 12 (Figure S4). For the phylum Bacteroidetes (Figure 4A), Ex increased the percentage of this bacteria for both LF fed (LF/Sed = 29.32±1.98% vs. LF/Ex = 40.67±6.19%) and HF fed mice (HF/Sed = 21.67±3.47% vs. HF/Ex = 29.80±5.23%; activity F(1,20) = 4.65, p = 0.044), with a trend for a diet effect (diet F(1,20) = 4.2, p = 0.054) but no interaction (F(1,20)  = 0.127, p = 0.73). Based on the post hoc test, HF/Sed was significantly different from LF/Ex (p = 0.044). For the phylum Firmicutes (Figure 4B), Ex decreased the percentage of this bacteria for both LF fed (LF/Sed = 67.97±2.27%, LF/Ex = 57.93±6.16%) and HF fed mice (HF/Sed = 76.83±3.77%, HF/Ex = 67.65±5.2%; activity F(1,20) = 4.38, p = 0.049), with a trend for a diet effect (diet F(1,20) = 4.09, p = 0.057) but no interaction (F(1,20) = 0.009, p = 0.93). Based on the post hoc test, there was a trend for difference between HF/Sed and LF/Ex (p = 0.051). Actinobacteria demonstrated significant differences as a result of diet and Ex (Figure 4C; diet F(1,20) = 4.85, p = 0.040; activity F(1,20) = 6.43, p = 0.02). Actinobacteria levels were highest in the LF/Sed group and significantly reduced by Ex (LF/Sed = 2.15±0.89%, LF/Ex = 0.15±0.15%). In the HF mice, Actinobacteria levels were fundamentally zero and there was an interaction (F(1,20) = 5.013,  = 0.037). Proteobacteria demonstrated a trend for elevation with HF diet (F(1,20) = 0.061), but not activity (Figure 4D). Tenericutes and Verrucomicrobia demonstrated no significant changes based on diet or Ex (Figure S4).

#### Quantitative polymerase chain reaction (q-PCR)

**Figure 5:**
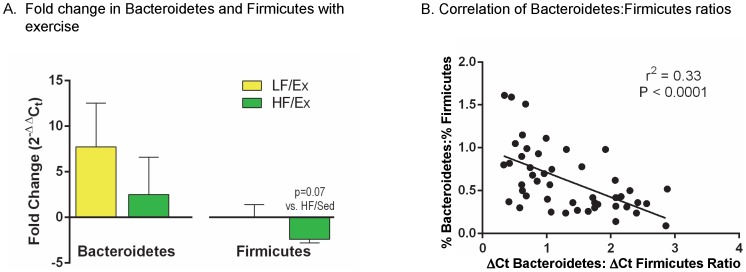
Diet and Activity Altered the Relative Level of Bacteroidetes and Firmicutes. A. The fold change in Bacteroidetes and Firmicutes was determined from the 2^−ΔΔCt^ values calculated from the ΔCt values generated by quantitative polymerase chain reaction (qPCR) using primers specific to each phyla (one-way ANOVA). B. Criterion validity of qPCR was examined by correlating the ΔCt-Bacteroidetes: ΔCt-Firmicutes ratio with the %-Bacteroidetes: %- Firmicutes ratios from sequencing. Data was analyzed by Pearson product-moment correlation coefficient and alpha level of p<0.05.

Quantitative PCR analysis of isolated fecal 16 s rRNA gene demonstrated no significant Ex effect on the content of the two major bacterial phyla, Bacteroidetes and Firmicutes, as demonstrated by the “fold change” in LF/Ex compared to LF/Sed and HF/Ex compared to HF/Sed using the 2^−ΔΔCt^ values (Figure 5A, statistical analysis based on comparison of ΔCt values). Using the primer and probe sets described by Armougom et al. [30] and Nadkarni et al. [31] (Table S1), the ratio of delta Ct values for Bacteroidetes and Firmicutes were calculated at weeks 0, 6 and 12 of the protocol. As shown in the Figure S5A, all groups had similar ΔCt ratios of Bacteroidetes:Firmicutes at week 0 (LF/Sed = 0.595±0.135, LF/Ex = 0.535±0.080, HF/Sed = 0.510±0.136, HF/Ex = 0.622±0.240). By 6 weeks, there was a trend for the HF diet mice to have a higher ΔCt ratio of Bacteroidetes:Firmicutes than the LF mice (LF/Sed = 0.672±0.072, LF/Ex = 0.730±0.117, HF/Sed = 1.03±0.20, HF/Ex = 1.127±0.344; diet F(1,20) = 3.22, p = 0.088). After 12 weeks, Ex significantly reduced the ΔCt ratios of Bacteroidetes:Firmicutes (LF/Sed = 2.293±0.165, LF/Ex = 1.543±0.177, HF/Sed = 2.068±0.219, HF/Ex = 1.232±0.257; activity F(1,20) = 14.61, p = 0.001), but diet did not have an effect (F(1,20 = 1.67, p = 0.21). Ex had a similar effect in both LF and HF mice (interaction F(1,20) = 0.44, p = 0.84).

Comparison of the Bacteroidetes to Firmicutes content in fecal samples was also calculated using sequencing data (Figure S5B). Comparable to the qPCR analysis, there was no demonstrable difference in the Bacteroidetes:Firmicutes ratio for any of the treatment groups at the start of the protocol. However, at 6 weeks, there was a significant reduction in the Bacteroidetes:Firmicutes ratio with HF diet (LF/Sed = 1.21±0.11, LF/Ex = 0.92±0.16, HF/Sed = 0.43±0.09, HF/Ex = 0.53±0.09; diet F(1,20) = 24.89, p<0.001), with no effect of activity or interaction. At week 12, Ex mice demonstrated a trend toward an increased Bacteroidetes:Firmicutes ratio in both the LF and HF diet mice (LF/Sed = 0.44±0.045, LF/Ex = 0.83±0.24, HF/Sed = 0.30±0.06, HF/Ex = 0.49±0.12; activity F(1,20) = 4.30, p = 0.051), but no diet effect or interaction was found.

As a test of the criterion validity of qPCR, a comparison was made between the qPCR and sequencing data. The ΔCt Bacteroidetes:ΔCt Firmicutes ratio determined by q-PCR and %Bacteroidetes:%Firmicutes ratio from sequencing was compared using a Pearson test on all data points from weeks 6 and 12. As shown in Figure 5B, there was a significant inverse correlation between the Bacteroidetes:Firmicutes ratios using delta Ct values and sequencing (r^2^ = 0.33, p<0.001). As expected, there was no relationship between the data points determined for week 0 (r^2^ = 0.005, p = 0.81).

**Figure 6:**
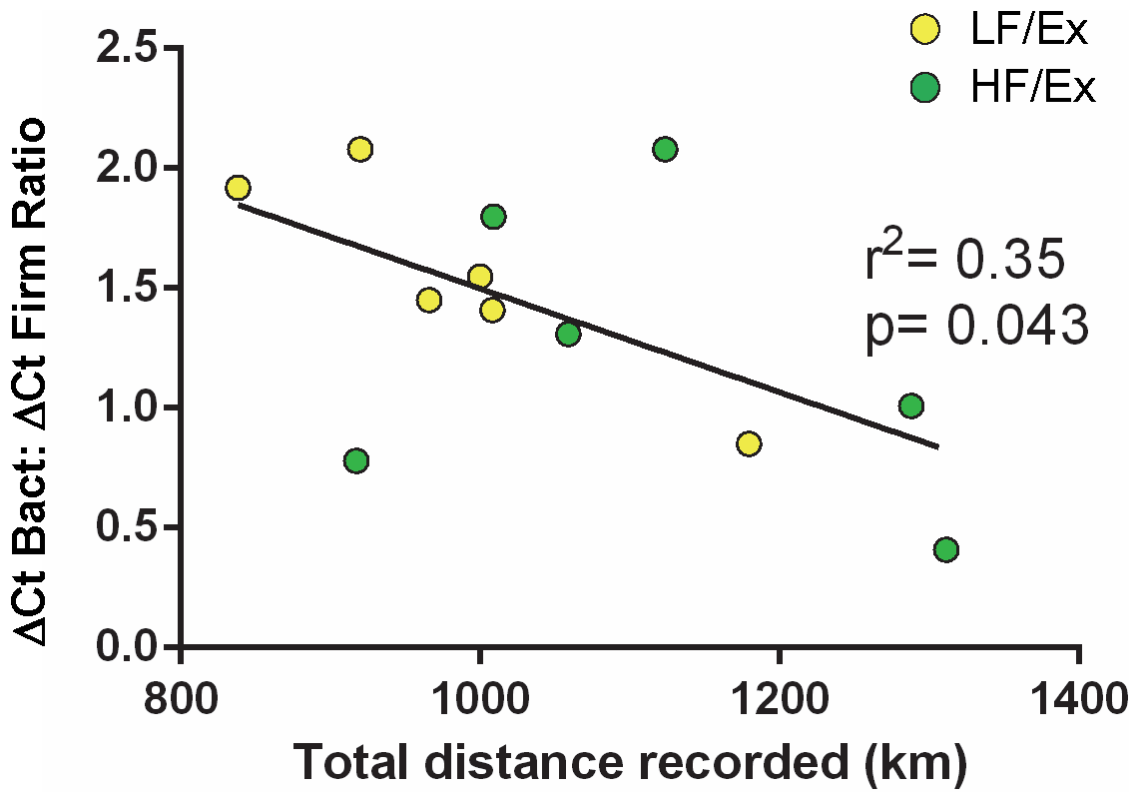
ΔCt Bacteroidetes: ΔCt Firmicutes Ratio Correlates with Exercise Distance. There was a significant, but modest inverse relationship between the ΔCt Bacteroidetes: ΔCt Firmicutes ratio and the distance recorded for the combined LF/Ex and HF/Ex mice. Data analyzed by Pearson product-moment correlation coefficient with an alpha level of p<0.05. n = 6 all groups.

To validate the activity effect on bacterial balance, total Ex distance was correlated to the resultant ΔCt ratio of Bacteroidetes:Firmicutes determined using qPCR (Figure 6). The ratio of delta Ct values for Bacteroidetes:Firmicutes correlated inversely with the Ex distance for the combined LF/Ex and HF/Ex groups (r^2^ = 0.35) based on a Pearson test (Pearson's r = −0.591, p = 0.043).

#### Diet and Activity-induced Changes in Fecal Bacteria

**Figure 7:**
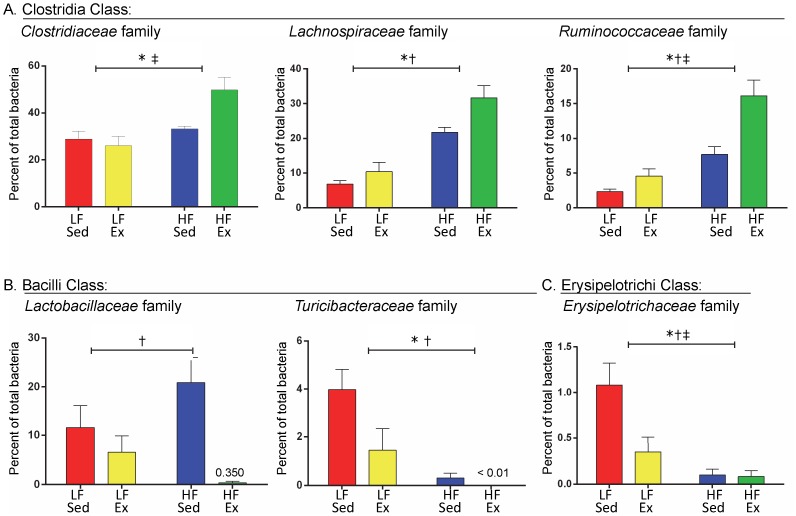
Diet and Activity Altered Firmicutes at the Family Level. Family level changes within the Firmicutes phyla were identified from sequencing the 16S rRNA gene from samples at week 12 of the diet and activity protocol. A. Three members of the Clostridia class were altered by the diet and activity protocol: *Clostridiaceae*, *Lachnospiraceae*, and *Ruminococcaceae*. B. Two members of the Bacilli class were altered by the diet and activity protocol: *Lactobacillaceae* and *Turicibacteraceae*. C. One family was altered within the *Erysipelotrichi* class by diet and activity: *Erysipelotrichaceae*. Data were analyzed by 2-way ANOVA with a Sidak post hoc test. Significant differences indicated as follows: “*” p<0.05 for diet effect, “†” p<0.05 activity effect and “‡” p<0.05 diet and activity interaction. n = 6 mice/group.

*Family Level Analysis*. Family-level changes within the major phyla induced by the diet and Ex protocol were revealed by the sequencing data. Within the Firmicutes phylum, there were significant changes in the classes Clostridia, Bacilli, and Erysipelotrichi (Figure 7). Three families of bacteria in the Clostridia class were altered by the diet and activity protocol: *Clostridiaceae*, *Lachnospiraceae*, and *Ruminococcaceae* (Figures 7A). High fat diet elevated the percentage of *Clostridiaceae* (diet F(1,20) = 13.76, p = 0.001), *Lachnospiraceae* (diet  = F(1,20) = 56.7, p<0.001) and *Ruminococcaceae* (diet F(1,20) = 37.38, p<0.001) in Sed and Ex mice. Ex significantly elevated the content of *Lachnospiraceae* (activity F(1,20) = 7.91, p = 0.011) and *Ruminococcaceae* (activity F(1,20) = 14.91, p = 0.001), with a tendency for an effect on *Clostridiacea* (activity F(1,20) = 3.17, p = 0.090). An interaction between diet and Ex was determined for *Clostridiaceae* (interaction F(1,20) = 6.54, p = 0.019; LF/Ex compared to HF/Ex, p = 0.0015) and *Ruminococcaceae* (interaction F(1,20) = 5.05, p = 0.036; activity had a greater effect in the HF fed mice compared to the LF mice). *Lachnospiraceae* had no interaction effect (F(1,20) = 1.72, p = 0.20).

The percentages of two different families within the class Bacilli were altered by the diet and activity protocol: *Lactobacillaceae* and *Turicibacteraceae* (Figures 7B). Ex decreased the percentage of *Lactobacillaceae* in LF and HF fed mice (F(1,20) = 11.27, p = 0.003), but there was no effect of diet (F(1,20) = 0.16, p = 0.69), although a trend for an interaction was detected (F(1,20) = 4.14, p = 0.055). HF/Sed mice demonstrated a bloom in *Lactobacillaceae* relative to the other treatment groups. *Turicibacteraceae* were significantly reduced by Ex in both LF and HF mice. High fat consumption decreased the percentage of *Turicibacteraceae* in the Sed and Ex groups (diet F(1,20) = 17.07, p<0.001; activity F(1,20) = 5.20, p = 0.034), with a trend for an interaction effect (F(1,20) = 3.23, p = 0.087).

A third class of Firmicutes, Erysipelotrichi, was also altered by diet and activity (Figure 7C). Within the class Erysipelotrichi, the percentage of the family *Erysipelotrichaceae* was reduced by both HF diet and Ex (diet F(1,20) = 17.15, p<0.001; activity F(1,20) = 6.17, p = 0.022), but Ex only exerted an effect in the LF fed mice (interaction F(1,20) = 5.64, p = 0.03; p = 0.016 LF/Sed greater than LF/Ex).

**Figure 8:**
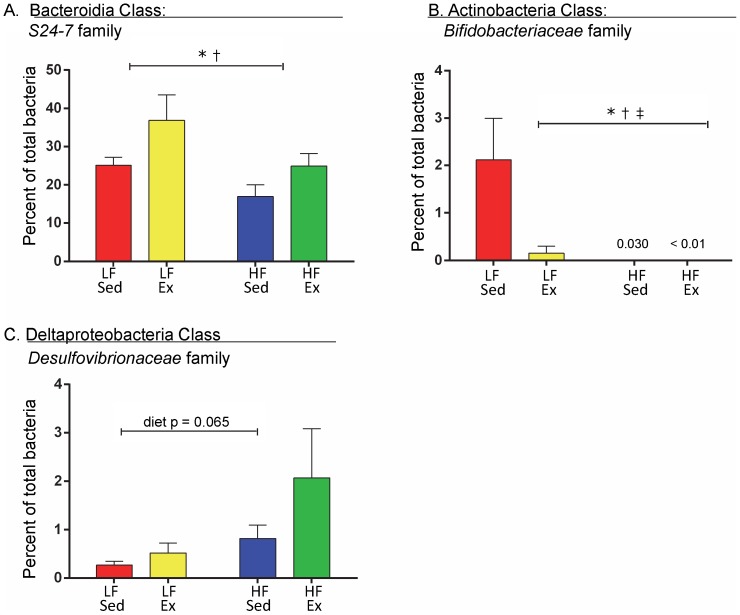
Diet and Activity Altered Families within the Bacteroidetes, Actinobacteria and Proteobacteria Phyla. Family level taxonomic groups were identified from sequencing 16S rDNA at week 12 of the diet and activity protocol. A. Within the phylum Bacteroidetes, the class Bacteroidia had one family that was altered by diet and activity: *S24-7*. B. Within the Actinobacteria phylum and class, one family was altered by diet and activity: *Bifidobacteriaceae*. C. Within the Proteobacteria phylum, the class Deltaproteobacteria had one family that demonstrated a trend toward an effect of diet and activity: *Desulfovibrionaceae*. Data were analyzed by 2-way ANOVA with a Sidak post hoc test. Significant differences indicated as follows: “*” p<0.05 for diet effect, “†” p<0.05 activity effect and “‡” p<0.05 diet and activity interaction. n = 6 mice/group.

Differences in the percentage of the phylum Bacteroidetes based on the diet and activity protocol could be explained primarily by differences in one class (Bacteroidia) and one family (*S24-7*, Figure 8A). High fat diet lowered the percentage of *S24-7* (F(1,20) = 5.89, p<0.025), but Ex increased it (F(1,20) = 5.63, p = 0.028) equally in LF and HF mice (F(1,20) = 0.204, p = 0.66). Differences in the percentage of the phylum Actinobacteria based on the diet and activity protocol could be explained by differences in only one class (Actinobacteria), one order (Bifidobacteriales) and one family (*Bifidobacteriaceae*, Figure 8B). Both HF diet and Ex lowered the percentage of *Bifidobacteriaceae* (diet F(1,20) = 6.45, p = 0.02; activity F(1,20) = 4.86, p = 0.039), with Ex affecting only the LF condition (interaction F(1,20) = 4.86, p = 0.039). Within the phylum Proteobacteria, a family in the Deltaproteobacteria class, *Desulfovibrionaceae*, demonstrated a trend toward a diet effect (Figure 8C) (diet F(1,20) = 3.81, P = 0.065), but there was no effect of activity or interaction. Other changes resulting from the diet and Ex protocol occurring at the class, order and family levels that did not reach statistical significance are provided in the Table S2.

## Discussion

This study examined the effect of voluntary Ex on the gut microbiota and the potential role of Ex-induced microbial changes in preventing HF-DIO in mice. Voluntary Ex, in and of itself, had significant effects on the relative balance of the major bacterial phyla, Bacteroidetes and Firmicutes, concurrent with prevention of DIO and normalization in glucose tolerance. In addition, Ex increased the Bacteroidetes:Firmicutes ratio in a manner that was proportional to the distance run. These results may suggest a possible microbial basis for Ex-mediated prevention of HF-DIO and may offer new insights regarding avenues to prevent DIO that could be useful to physically-challenged individuals who are not able to voluntarily Ex to manage their weight.

Balance between Bacteroidetes and Firmicutes has been reported to change with obesity as well as with the proportion of lean body mass [11], [13]. Bacteroidetes in the gut microbiome is directly related to lean body mass: levels are reduced with obesity [8], [13], [36] and elevated when obese individuals lose weight [8]. While many rodent and human studies demonstrate an inverse relationship between obesity and the proportion of Bacteroidetes, there are reports in rats and humans that demonstrate an increase in the relative proportion of Bacteroidetes in response to a HF diet [37]. Our study supports that prevention of weight gain, in this case by Ex, is associated with a relative increase in Bacteroidetes. Alternately, fiber consumption [38] and the weight loss-promoting effects of mono-unsaturated fatty acids (oleic acid) and polyphenols are also associated with a rise in Bacteroidetes [39], [40]. Two recent studies suggest that the richness and diversity of the gut microbiota inversely correlates with obesity [41], [42]. Our results, in part, support this observation since Ex elevated the diversity of the gut microbiota, but only in the HF group.

The exact effect of HF consumption on the microbiome is likely dependent on the relative proportion of saturated, mono- and poly-unsaturated fat in the diet. In the current study, lard was the fat source with 32% saturated, 35.9% mono- and 32% poly-unsaturated fats. Huang, et al. reported that diets rich in polyunsaturated fat (PUFA;74%) elevated levels of Firmicutes, Tenericutes, Proteobacteria, and Actinobacteria relative to the LF controls [43]. The PUFA diet also increased inflammatory gene activation in mesenteric fat compared to lard or milk fat containing diets. However, in IL-10 knockout mice, a line genetically susceptible to intestinal inflammation, milk fat (58% saturated fat) produced a bloom in the Proteobacteria, *Bilophila wadsworthia*, which resulted in a doubling of the incidence rate for spontaneous colitis [44]. Interestingly, the HF lard diet used in this study also induced an increase in Proteobacteria primarily resulting from an increase in the order Desulfovibrionales, to which *B. wadworthia* belongs.

In the current study, Ex significantly increased the relative proportion of butyrate-producing bacteria such as Bacteroidales *S24-7*. High-fat sedentary mice had the lowest level of *S24-7* family compared to the other treatment groups and the level was elevated by Ex. Exercise also led to an increase in butyrate-producing Firmicutes in fecal samples: *Clostridiaceae*, *Lachnospiraceae* and *Ruminococcaceae*. These bacteria are all members of Clostridiales order and are also linked to butyrate production [45]. Matsumoto et al. demonstrated that voluntary running significantly increased butyrate producing cecal bacteria (members of Clostridiales order) as well as elevated the level of cecal *n*-butyrate in non-obese rats [21]. Butyrate production in the large intestines is associated with intestinal epithelial cell health and the production of heat shock protein 70 (Hsp70) [46], [47]. Hsp70 has been reported to maintain the functional and structural properties of intestinal epithelial cells in response to bacterial challenge, ischemia, and other environmental stresses, including Ex [47]–[50]. Previous reports indicated that epithelial cell integrity can be compromised by HF induced changes in barrier function, thus producing low grade systemic inflammation [51], [52]. Butyrate stimulates epithelial cell Hsp70 production, which can provide structural and functional stability to cells under the stress of HF dietary intake.

Exercise also reduced the percentage of the family *Lactobacillaceae* in the HF mice, dropping it from greater than 20% in the HF/Sed mice to 0.35% in the HF/Ex mice. This dramatic reduction with Ex suggests an important role for *Lactobacillaceae* in sedentary lifestyle-associated weight gain. Lactobacilli are often used as probiotic agents. Depending on the *Lactobacillus* species and experimental model, the reported relationship between detectable *Lactobacillus* spp and the effects of *Lactobacillus* on weight gain and obesity can be quite different. *Lactobacillus* spp are reported to be elevated in obese children [53]. In obese adults, *Lactobacillus reuteri* is elevated, but *L. paracasei* and *L. plantaru*m are reported to be reduced [54]. *Lactobacillus* spp given as probiotic therapy have dose-dependent effects with regards to weight changes [55], [56] whereas *L. plantarum* has been reported to improve glucose sensitivity in response to a HF diet [57], [58].

The central questions raised by this study are, how does Ex alter the gut microbiota and does this contribute to the prevention of HF-DIO? Whether Ex operates to prevent HF-DIO through direct changes in the gut microbiota is an intriguing question because the answer could lead to new probiotics or prebiotics that provide for some of the benefits of Ex. The presence of a gut microbiota is known to help break down ingested food and likely allows for additional nutrient extraction not available to a germ free host [10], [59]. Turnbaugh et al. [8] showed that a gut microbiota associated with obesity in ob/ob mice that was depleted in Bacteroidetes and enriched in Firmicutes was capable of extracting more energy from the diet compared to the microbiota from their lean counterparts. In particular, the presence of a gut microbiota has been shown to increase uptake of SCFAs in the colon [60], [61].

Three factors determine the bacterial production of SCFA in the colon: the gut microbiota, availability of substrates, and intestinal transit time [60]. Based on our findings, changes in the gut microbiota with Ex could result in altered SCFA production and/or utilization. While gut transit time for substrates into and within the colon is also important [60], this study and that of Choi, et al. did not detect an effect of Ex on gut transit [22]. Several studies have shown that Ex is associated with increased gut motility [18], [62], [63]. We examined, primarily, small intestinal transit, and detected no change between the sedentary and Ex mice. If Ex had changed small intestinal transit, it could significantly enhance nutrient delivery to the cecum and colon, thereby changing microbial balance simply due to nutrient availability.

The amount of nutrients ingested could also impact bacterial content. In this study, Ex increased 24-hour caloric intake compared to Sed mice. Thus, microbial changes detected in the HF/Ex group beyond that of the HF/Sed could be related to even greater fat intake compared to HF/Sed or to an independent effect of Ex. A recent study by Simoes et al. demonstrated a change in the proportion of Bacteroidetes in monozygotic twins who were discordant with respect to diet [38]. They reported an inverse relationship between energy intake and the proportion of Bacteroidetes. This inverse relationship is interesting in light of the current results since in this study Ex increased both energy consumption and the proportion of Bacteroidetes. Acute bouts of exercise have also been shown to cause suppression of appetite during and immediately afterward with short-term negative energy balance, followed by increased appetite and positive energy balance [64], [65]. These changes could lead to altered feeding patterns in Ex mice that could affect the gut microbiota.

An Ex-induced gut microbiota may increase fatty acid oxidation and utilization or decrease fat storage in the intestine. Exercise up-regulates AMP-activated protein kinase (AMPK) [66], a molecular sensor of both cellular and systemic energy states. This pathway is also up-regulated in germ free (GF) mice and is believed to play a critical role in resistance to HF-DIO in this model [61]. Whether Ex only directly activates the AMPK system or also works through changes in the microbiota is still not clear, but activation of the AMPK system has been reported to be protective from hyperglycemia and type-2 diabetes and our findings related to improved OGT in HF/Ex mice compared to HF/Sed support this. We must also consider that Ex might act independently of the gut microbiota, by increasing energy utilization despite consumption of a HF diet.

Another way in which Ex could exert a potentially beneficial effect through the gut microbiota is by suppressing inflammation. Exercise might prevent HF-DIO by blocking HF feeding-associated inflammation. Obesity and HF diets are linked to increased markers of endotoxemia and inflammation [67]. IL-6, a key myokine that is up-regulated by muscle contraction, suppresses TNF-α and the inflammatory response [68], [69]. Exercise could regulate the gut microbiota through IL-6-mediated immunomodulation.

One of the limitations of this study is that bacterial changes were only measured in fecal samples. Differences in the composition of the gut microbiota have been shown as one moves from the epithelial surface out into the lumen of the intestine [70]. Different bacterial profiles may have been obtained if luminal scrapings or cecal contents had been examined. Another possible limitation is that while the correlation between the Bacteroidetes:Firmicutes ratios calculated using qPCR and sequencing is significant (p<0.001), it is a weak relationship (r^2^ = 0.33). This may be due to differences in the two techniques, including the methods of quantification (cycle number versus database-matching), universal primer sets used, and the specificity and inclusiveness of sequencing compared to qPCR. However, it can be seen in the Figure S4A and S4B that qPCR and sequencing detected similar changes with development as well as dietary and activity interventions. This study did not identify bacteria below the taxonomic level of family. Changes at the genus and species level would be interesting in terms of comparisons to species with known metabolic or pathological functions. While food intake and gut transit were explored, energy extraction from the diet and changes in indicators of endotoxemia and inflammation were not included. These mechanisms are important to examine in terms of understanding whether the changes in the gut microbiota demonstrated with Ex are part of the anti-inflammatory, beneficial effect of Ex in preventing HF-DIO or whether they are coincidental with only a direct effect of Ex on energy balance and inflammation. Given the complex nature of the gut microbiota's interactions with the host's immune and metabolic systems, teasing out the contribution of Ex to these interactions will be difficult.

### Summary/Conclusions

Exercise alters the gut microbiota in mice on both a LF and HF diet and normalizes major phylum-level changes for mice on the HF diet; furthermore, the volume of exercise (total distance run) inversely correlates with the Bacteroidetes:Firmicutes ratio. The Ex-induced gut microbiota in both LF and HF conditions are different than their sedentary counterparts and yet, also different from each other. At the taxonomic level of bacterial family, HF diet induced blooms in some of the major constituent groups within Firmicutes such as *Lactobacillaceae*, *Lachnospiraceae*, and *Ruminococcaceae* and a decrease in the major constituent group of Bacteroidetes, *S24-7*. Exercise in the HF condition prevented some of the dietary changes, such as with *Lactobacillaceae* and S24-7, but intensified the blooms seen with HF feeding in others such as with *Lachnospiraceae*, and *Ruminococcaceae*. Further exploration of the gut microbiota changes induced by Ex may allow for exploitation of this effect and the development of treatments for obesity and dysbiosis associated with high fat intake.

## Supporting Information

Figure S1 **Identification of Amplicon Size for Bacteroidetes, Firmicutes and the Universal Primer Sets.**
(PDF)

Figure S2 **Diet and Activity Changed Bacterial Profiles Determined by T-RFLP and PCA.**
(PDF)

Figure S3 **Dendrogram Analysis of Diet and Activity Effects.**
(PDF)

Figure S4 **Diet and Activity Altered Major and Minor Bacterial Phyla.**
(PDF)

Figure S5 **Changes in the Bacteroidetes:Firmicutes Ratio Determined by qPCR and Sequencing.**
(PDF)

Table S1 **Primer and probe sets for Bacteroidetes and Firmicutes qPCR.**
(PDF)

Table S2 **Effect of diet and exercise on bacterial families with total contributions between 0% and 1.25%.**
(PDF)

File S1 **Commands used in the analysis of the sequencing dataset.**
(PDF)
