# Supplementary material for: Exercise Prevents Weight Gain and Alters the Gut Microbiota in a Mouse Model of High Fat Diet-Induced Obesity
Source: PLoS One. 2014 Mar 26;9(3):e92193. doi: 10.1371/journal.pone.0092193 (PMC3966766; doi:10.1371/journal.pone.0092193)
Supplement: Figure S1 — Identification of Amplicon Size for Bacteroidetes, Firmicutes and the Universal Primer Sets. (PDF) [file pone.0092193.s001.pdf]

Data Supplement, Figure S1.

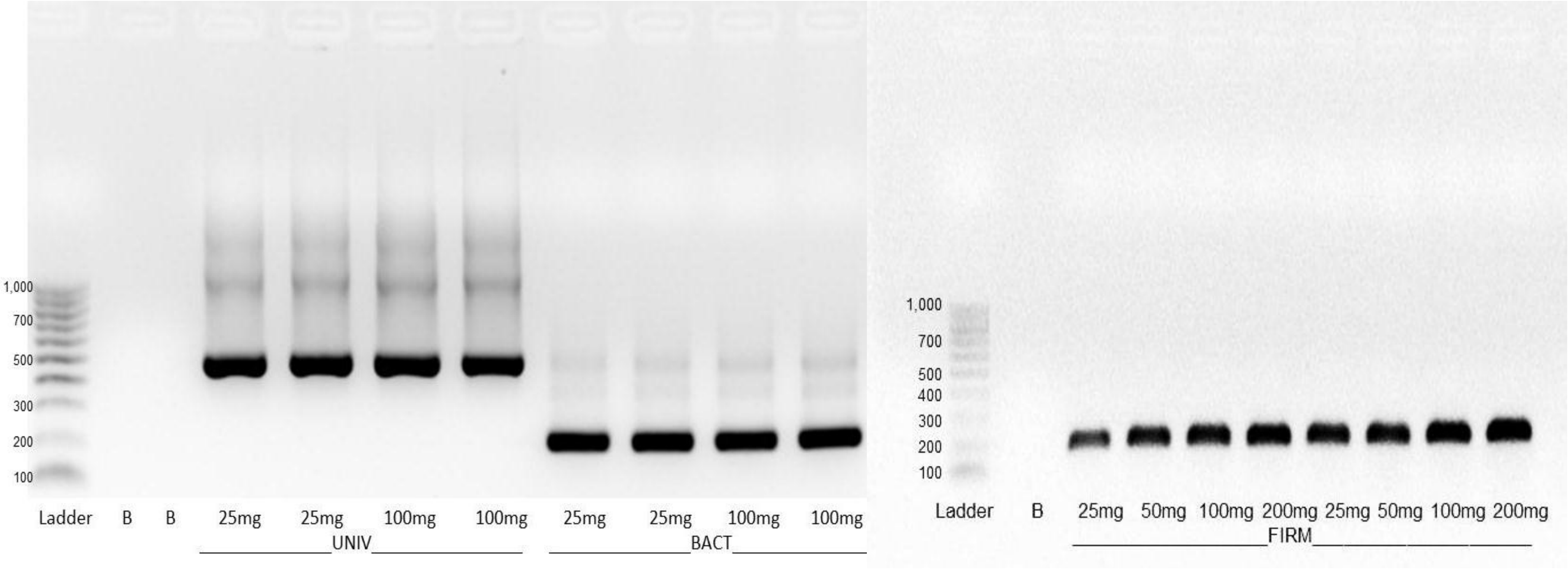

Data supplement- Figure S1. *Identification of Amplicon Size for Bacteroidetes, Firmicutes and the Universal Primer Set.* Scan of two agrose gels showing the amplicon size for the universal (UNIV), Bacteroidetes (BACT), and Firmicutes (FIRM) primers used for quantitative polymerase chain reaction (qPCR) assays. The size of the universal primer amplicon was 466 base pairs, the size of the Bacteroidetes amplicon was 184 base pairs and the size of the Firmicutes primer amplicon was 179 BP. The methods for the Bacteroidites and Firmicutes primers were modified from the methods of Armougom et al.[1] and methods for the universal primers were modified from the methods of Nadkarni et al.[2] The primer sequences are shown in Supplemental Table 1. A total of 200 nM of the forward and reverse primers were used for each of the 3 assays and samples were at 20ng/μL but at varying amounts of total DNA. The final volume of all assays was 25μL. All qPCR runs were as follows: pre-amplification stage 95 °C for 10 min and (40 cycles at 95 °C x15 seconds, 60 °C x 1 minute) for each cycle and were run for 30 cycles.

Bibliography

1. Armougom F, Henry M, Vialettes B, Raccah D, Raoult D (2009) Monitoring bacterial community of human gut microbiota reveals an increase in Lactobacillus in obese patients and Methanogens in anorexic patients. PLoS One 4: e7125.

2. Nadkarni MA, Martin FE, Jacques NA, Hunter N (2002) Determination of bacterial load by real-time PCR using a broad-range (universal) probe and primers set. Microbiology 148: 257-266.
