# Supplementary material for: Exercise Prevents Weight Gain and Alters the Gut Microbiota in a Mouse Model of High Fat Diet-Induced Obesity
Source: PLoS One. 2014 Mar 26;9(3):e92193. doi: 10.1371/journal.pone.0092193 (PMC3966766; doi:10.1371/journal.pone.0092193)
Supplement: Figure S2 — Diet and Activity Changed Bacterial Profiles Determined by T-RFLP and PCA. (PDF) [file pone.0092193.s002.pdf]

Data Supplement, Figure S2

A. Examples of raw electropherograms

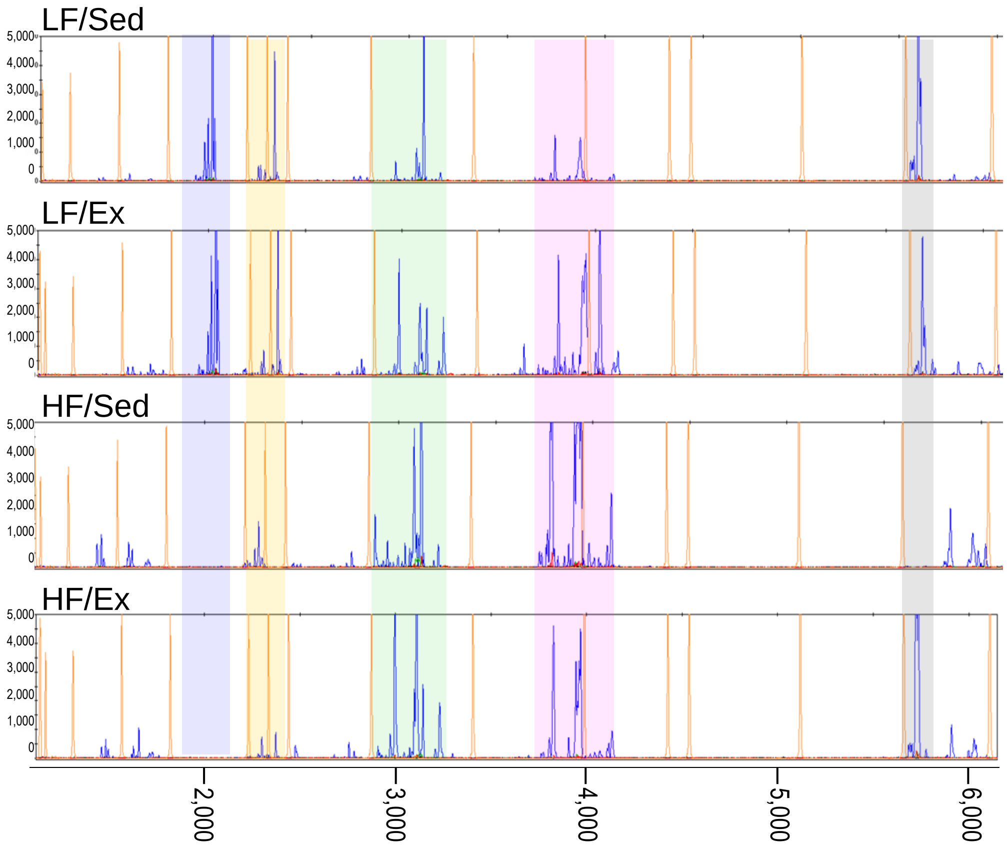

B. Principal coordinate analysis of week 0 samples

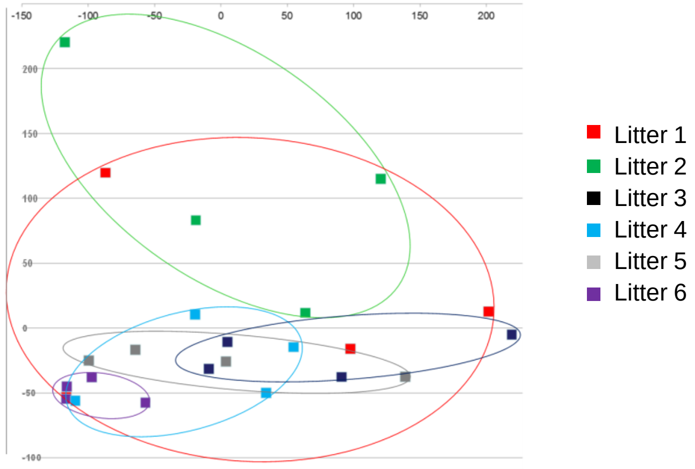

IPCA-1= 37.4%, IPCA-2 17.5% (55%), IPCA-3 9.6% (65%), IPCA-4 8.6% (73.2%)

C. Principal coordinate analysis of week 12 samples

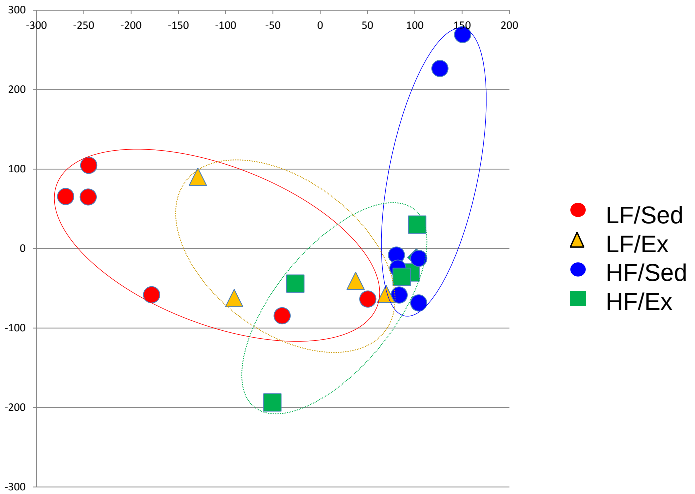

IPCA 1=80.8% (80.8%), IPCA 2=11.2% (92%) and IPCA 3=8% (100%)

Data supplement- Figure S2. *Diet and Activity Changed Bacterial Profiles Determined by T-RFLP and PCA.* Terminal-restriction fragment length polymorphism analysis (T-RFLP) was performed on 16S rDNA isolated from fecal samples collected at weeks 0 and 12 of the diet and exercise protocol. A. Examples of representative raw electropherogram are shown from each of the treatment groups [(low-fat/sedentary (LF/Sed), low-fat/exercise (LF/Ex), high-fat/sedentary (HF/Sed) and high-fat/exercise (HF/Ex)] at week 12 of the diet and exercise protocol. The X-axis indicates the length of the fragments in molecular weight and the Y-axis indicates abundance. Orange spikes on the electropherograms indicate molecular weight markers added to allow for standardization and blue peaks indicate fragments from samples. Colored vertical bands (blue, yellow, green, pink and gray) were added to show obvious areas within the electropherograms that differed between the groups. B. Principal coordinate analysis (PCA) was performed at week 0 of the diet and exercise protocol. The samples are identified by litter (6 litters, 1-6, of 4-8 mice/litter) based on the key and some clustering was detected based on litter. No clustering or distribution based on diet and exercise pattern was evident at week 0. C. PCA was performed at week 12 of the diet and exercise protocol. The samples are identified by diet and exercise group and ovals were drawn to indicate clustering.
