# Supplementary material for: Exercise Prevents Weight Gain and Alters the Gut Microbiota in a Mouse Model of High Fat Diet-Induced Obesity
Source: PLoS One. 2014 Mar 26;9(3):e92193. doi: 10.1371/journal.pone.0092193 (PMC3966766; doi:10.1371/journal.pone.0092193)
Supplement: Figure S3 — Dendrogram Analysis of Diet and Activity Effects. (PDF) [file pone.0092193.s003.pdf]

Data Supplement, Figure S3

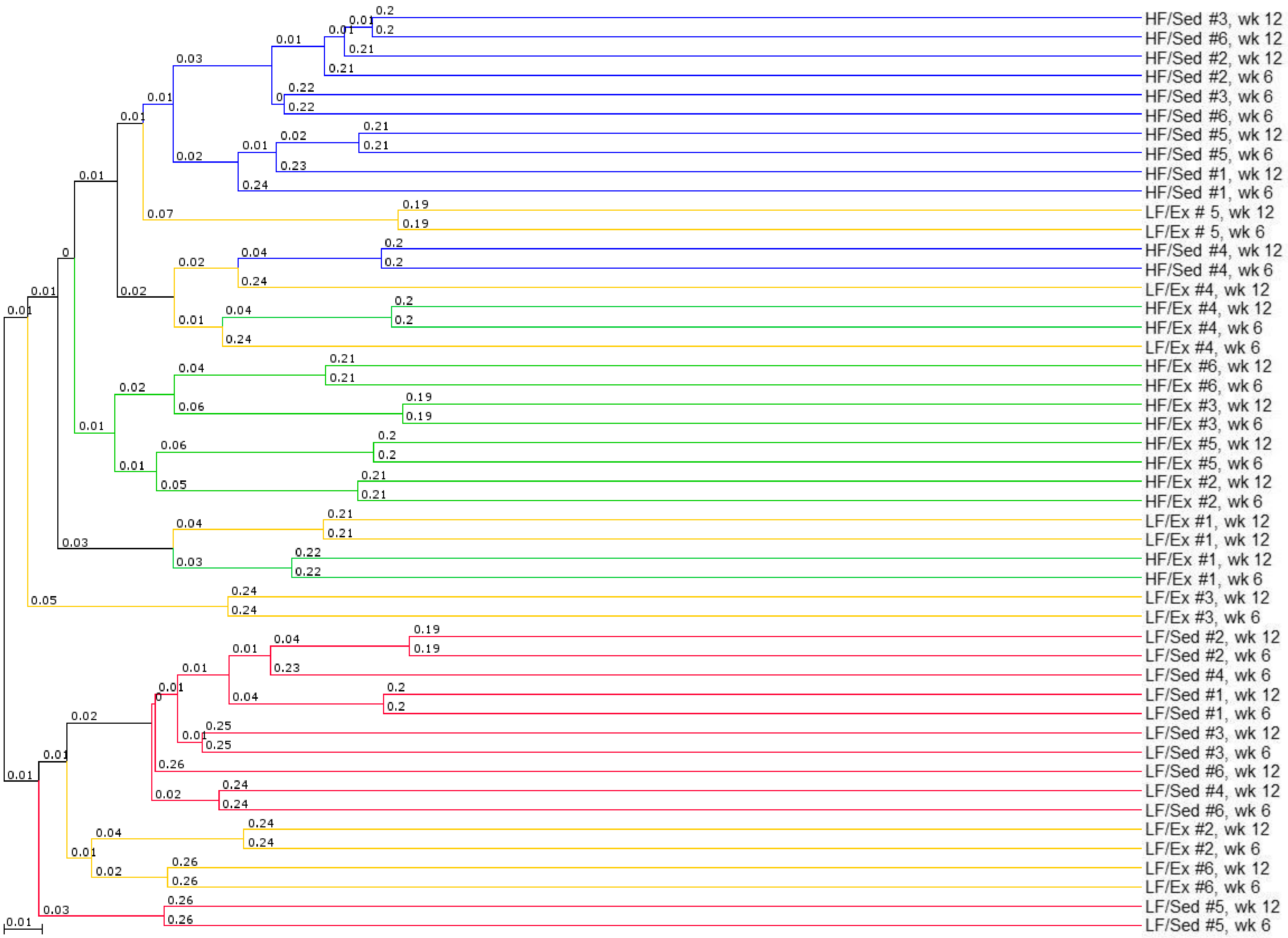

Data Supplement- Figure S3. *Dendrogram Analysis of Diet and Activity Effects*. A dendrogram created using the weighted UniFrac distance matrix generated from sequencing fecal 16S rDNA samples from mice at week 12 of the protocol demonstrates clustering of the samples by diet and activity. The labels on the right Y-axis represent the group, litter number and week when the sample was taken. Numbers associated with line junctions represent the distance of related samples based on the weighted UniFrac distance matrix.
