# Supplementary material for: Exercise Prevents Weight Gain and Alters the Gut Microbiota in a Mouse Model of High Fat Diet-Induced Obesity
Source: PLoS One. 2014 Mar 26;9(3):e92193. doi: 10.1371/journal.pone.0092193 (PMC3966766; doi:10.1371/journal.pone.0092193)
Supplement: Figure S4 — Diet and Activity Altered Major and Minor Bacterial Phyla. (PDF) [file pone.0092193.s004.pdf]

Data Supplement, Figure S4:

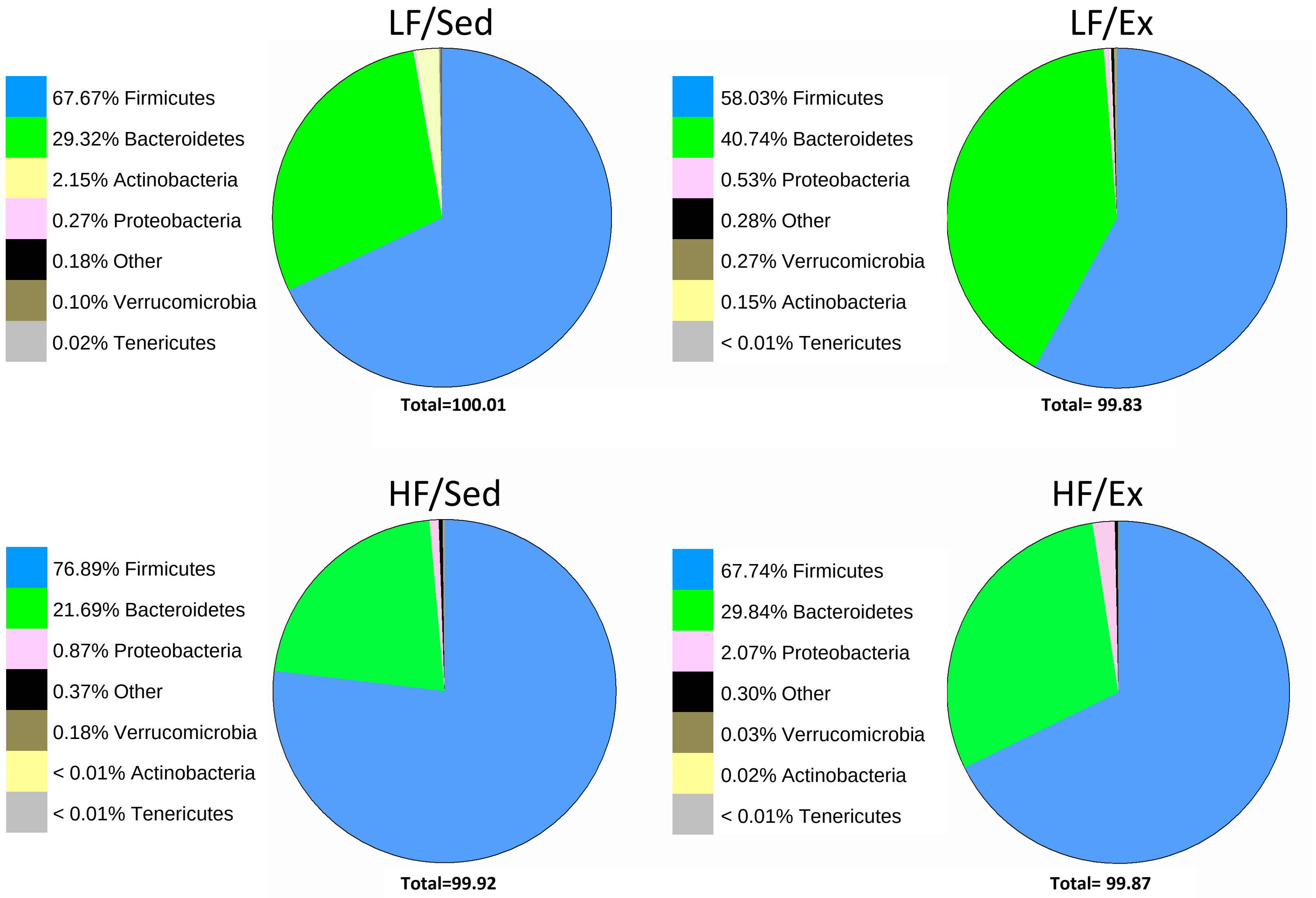

Data Supplement- Figure S4. *Diet and Activity Altered Major and Minor Bacterial Phyla*. Sequencing identified 6 phyla of bacteria in fecal samples based on analysis of the 16S rRNA gene. Note that not all of the minor phyla were represented in each of the diet and activity groups. n= 6 mice/group.
