# Supplementary material for: Exercise Prevents Weight Gain and Alters the Gut Microbiota in a Mouse Model of High Fat Diet-Induced Obesity
Source: PLoS One. 2014 Mar 26;9(3):e92193. doi: 10.1371/journal.pone.0092193 (PMC3966766; doi:10.1371/journal.pone.0092193)
Supplement: Figure S5 — Changes in the Bacteroidetes:Firmicutes Ratio Determined by qPCR and Sequencing. (PDF) [file pone.0092193.s005.pdf]

Data Supplement, Figure S5

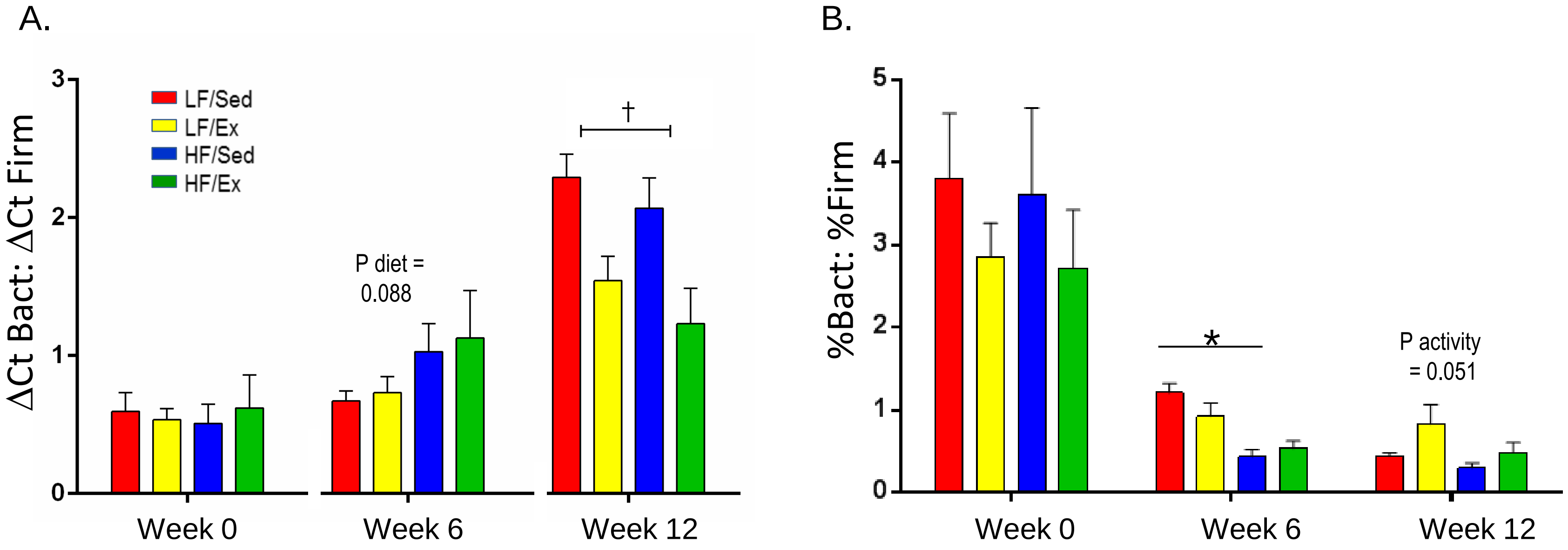

Data supplement- Figure S5. *Changes in the Bacteroidetes:Firmicutes Ratio Determined by qPCR and Sequencing.* A. The ratio of the ΔCt-Bacteroidetes:ΔCt-Firmicutes from qPCR at weeks 0, 6 and 12. and B. %-Bacteroidetes: %-Firmicutes from sequencing at weeks 0, 6 and 12. Data are presented as mean ± SEM. Significant differences are indicated as follows: "*" $P < 0.05$ for diet effect, "†" $P < 0.05$ activity effect and "‡" $P<0.05$ diet and activity interaction.
