# Supplementary material for: Exercise Prevents Weight Gain and Alters the Gut Microbiota in a Mouse Model of High Fat Diet-Induced Obesity
Source: PLoS One. 2014 Mar 26;9(3):e92193. doi: 10.1371/journal.pone.0092193 (PMC3966766; doi:10.1371/journal.pone.0092193)
Supplement: Table S1 — Primer and probe sets for Bacteroidetes and Firmicutes qPCR. (PDF) [file pone.0092193.s006.pdf]

Data Supplement, Table S1. Primer and probe sets for Bacteroidetes and Firmicutes qPCR

Target	Forward primer	Reverse primer	Probe
Bacteroidetes 16S rDNA	AGCAGCCGCGGTAAT	CTAHGCATTTCACCGCTA	6FAM-GGGTTTAAAGGG-MGBNFQ
Firmicutes 16S rDNA	GTCAGCTCGTGTCGTGA	CCATTGTAKYACGTGTGT	6VIC-GTCAANTCATCATGCC-MGBNFQ
Universal 16S rDNA	TCCTACGGGAGGCAGCAGT	GGACTACCAGGGTATCTAATCCTGTT	6FAM-CGTATTACCGCGGCTGCTGGCAC-3TAMRA

The complete sequence for the 3 primer and probe sets used for quantitative polymerase chain reaction (qPCR) assays are shown. The Bacteroidetes and Firmicutes primers were based on the methods of Armougom et al. and the universal primers were based on the methods of Nadkarni et al.
