# Supplementary material for: Exercise Prevents Weight Gain and Alters the Gut Microbiota in a Mouse Model of High Fat Diet-Induced Obesity
Source: PLoS One. 2014 Mar 26;9(3):e92193. doi: 10.1371/journal.pone.0092193 (PMC3966766; doi:10.1371/journal.pone.0092193)
Supplement: Table S2 — Effect of diet and exercise on bacterial families with total contributions between 0% and 1.25%. (PDF) [file pone.0092193.s007.pdf]

Data Supplement, Table S2. Effect of diet and exercise on bacterial families with total contributions between 0% and 1.25%

Phyla	Class	Order	Family	LF/Sed	LF/Ex	HF/Sed	HF/Ex	Notes
Firmicutes	Other	Other	Other	A	A	S (5/6) ND: 11	S (5/6) ND: 16	Sed > Ex [a]
Firmicutes	Clostridia	Clostridiales	Other	A	S (5/6) ND: 14	A	A	NS
Firmicutes	Clostridia	Clostridiales	Peptococcaceae	N	N	S (1/6) D: 23	S (1/6) D: 24	NS Litter F: 23 & 24
Firmicutes	Clostridia	Clostridiales	Dehalobacteriaceae	S (1/6) D: 1	S (2/6) D: 2,14	S (5/6) ND: 3	S (4/6) ND: 4,16	LF < HF [b] Litter A: 1, 2, 3 & 4 Litter D: 14 & 16
Firmicutes	Clostridia	Coriobacteriales	Coriobacteriaceae	A	A	A	A	LF < HF [c]
Firmicutes	Bacilli	Other	Other	S (4/6) ND: 9,13	S (4/6) ND: 2,10	A	N	LF/Ex > HF/Ex [d] HF/Sed > HF/Ex [d] Litter C: 9 & 10
Firmicutes	Bacilli	Lactobacillales	Other	N	S (1/6) D: 18	S (3/6) D: 3,11,15	N	LF/Sed < HF/Sed [e] HF/Sed > HF/Ex [e]
Firmicutes	Bacilli	Lactobacillales	Enterococcaceae	N	N	S (2/6) D: 19,23	S (1/6) D: 20	NS Litter E: 19 & 20
Tenericutes	Mollicutes	Anaeroplasmatales	Anaeroplasmataceae	N	S (3/6) D: 2,10,14	N	N	NS
Bacteroidetes	Bacteroidia	Bacteroidales	[Odoribacteraceae]	N	S (2/6) D: 6,18	N	S (1/6) D: 12	NS

Within a diet and exercise group, some bacteria were detected in all six mice (A: always), some bacteria were not detected in any mice (N: Never), and other bacteria were detected in 1-5 mice in the group (S: Sometimes). For S, the number of mice in which the bacterium was either detected (D) or not detected (ND) is indicated as a fraction ("*/6"). The identifying number for each mouse is also denoted below. The mouse litters were as follows: Litter A: mice #1-4, Litter B: mice #5-8, Litter C: mice #9-12, Litter D: mice #13-16, Litter E: mice #17-20, and Litter F: mice #21-24. Assignment of mice to treatment groups were as follows: LF/Sed: mice #1, 5, 9, 13, 17, 21; LF/Ex: mice #2, 6, 10, 14, 18, 22; HF/Sed: mice #3, 7, 11, 15, 19, 23; HF/Ex: mice #4, 8, 12, 16, 20, 24. NS: not significant by 2-way ANOVA; [a]Main effect exercise $F(1,20)=7.118$, $P=0.015$; [b]Main effect diet $F(1,20)=6.923$, $P=0.016$; [c]Main effect diet $F(1,20)=17.361$, $P<0.001$; [d]Interaction $F(1,20)=7.313$, $P=0.014$; [e]Interaction $F(1,20)=4.623$, $P=0.044$.
