# Supplementary material for: Exercise Prevents Weight Gain and Alters the Gut Microbiota in a Mouse Model of High Fat Diet-Induced Obesity
Source: PLoS One. 2014 Mar 26;9(3):e92193. doi: 10.1371/journal.pone.0092193 (PMC3966766; doi:10.1371/journal.pone.0092193)
Supplement: File S1 — Commands used in the analysis of the sequencing dataset. (PDF) [file pone.0092193.s008.pdf]

Supplementary Text S1: Commands used in the analysis of the sequencing dataset

#SampleID	BarcodeSequence	LinkerPrimerSequence	Mouse	Sort	Diet	Lifestyle	Litter	Week	Diet.LifeStyle	Diet.Litter	Diet.Week	LifeStyle.Litter	LifeStyle.Week	Litter.Week	Diet.LifeStyle.Week	shannon	initialwt	finalwt	fatpad	DistRun	OGTT	Description
Evans.1.1	GGAATTATCGGT	CCGGACTACHVGGGTWTCTAAT	1	1	LowFat	Sedentary	A	1	LowFat.Sedentary	LowFat.A	LowFat.1	Sedentary.A	Sedentary.1	A.1	LowFat.Sedentary.1	4.415	18.2	28.5	0.925	NA	22328	Evans.1.1
Evans.1.5	GGCAAATACACT	CCGGACTACHVGGGTWTCTAAT	5	2	LowFat	Sedentary	B	1	LowFat.Sedentary	LowFat.B	LowFat.1	Sedentary.B	Sedentary.1	B.1	LowFat.Sedentary.1	3.929	19.1	28.2	0.744	NA	20400	Evans.1.5
Evans.1.9	GCTTAGATGTAG	CCGGACTACHVGGGTWTCTAAT	9	3	LowFat	Sedentary	C	1	LowFat.Sedentary	LowFat.C	LowFat.1	Sedentary.C	Sedentary.1	C.1	LowFat.Sedentary.1	4.155	16.1	25.2	0.576	NA	22575	Evans.1.9
Evans.1.13	CTCTCTCACTTG	CCGGACTACHVGGGTWTCTAAT	13	4	LowFat	Sedentary	D	1	LowFat.Sedentary	LowFat.D	LowFat.1	Sedentary.D	Sedentary.1	D.1	LowFat.Sedentary.1	3.505	18.7	27.6	0.824	NA	25898	Evans.1.13
Evans.1.17	CTAGGATCACTG	CCGGACTACHVGGGTWTCTAAT	17	5	LowFat	Sedentary	E	1	LowFat.Sedentary	LowFat.E	LowFat.1	Sedentary.E	Sedentary.1	E.1	LowFat.Sedentary.1	4.228	17.7	26.2	0.489	NA	18818	Evans.1.17
Evans.1.21	CAATCGGCTTGC	CCGGACTACHVGGGTWTCTAAT	21	6	LowFat	Sedentary	F	1	LowFat.Sedentary	LowFat.F	LowFat.1	Sedentary.F	Sedentary.1	F.1	LowFat.Sedentary.1	5.07	18.5	26.9	0.486	NA	17880	Evans.1.21
Evans.1.2	CATCAAGCATAG	CCGGACTACHVGGGTWTCTAAT	2	7	LowFat	Exercised	A	1	LowFat.Exercised	LowFat.A	LowFat.1	Exercised.A	Exercised.1	A.1	LowFat.Exercised.1	4.723	17.9	26	NA	1046	19313	Evans.1.2
Evans.1.6	GTCATGCTCCAG	CCGGACTACHVGGGTWTCTAAT	6	8	LowFat	Exercised	B	1	LowFat.Exercised	LowFat.B	LowFat.1	Exercised.B	Exercised.1	B.1	LowFat.Exercised.1	4.237	19	24.9	0.485	1056	18188	Evans.1.6
Evans.1.10	AAGACGTAGCGG	CCGGACTACHVGGGTWTCTAAT	10	9	LowFat	Exercised	C	1	LowFat.Exercised	LowFat.C	LowFat.1	Exercised.C	Exercised.1	C.1	LowFat.Exercised.1	4.047	18.4	25.8	0.333	976.7	29040	Evans.1.10
Evans.1.14	ATTGCAAGCAAC	CCGGACTACHVGGGTWTCTAAT	14	10	LowFat	Exercised	D	1	LowFat.Exercised	LowFat.D	LowFat.1	Exercised.D	Exercised.1	D.1	LowFat.Exercised.1	3.764	18.2	24.7	0.578	1406.8	18900	Evans.1.14
Evans.1.18	GATGACCCAAAT	CCGGACTACHVGGGTWTCTAAT	18	11	LowFat	Exercised	E	1	LowFat.Exercised	LowFat.E	LowFat.1	Exercised.E	Exercised.1	E.1	LowFat.Exercised.1	4.755	19	25.6	0.539	1119.899	23865	Evans.1.18
Evans.1.22	AACACTCGATCG	CCGGACTACHVGGGTWTCTAAT	22	12	LowFat	Exercised	F	1	LowFat.Exercised	LowFat.F	LowFat.1	Exercised.F	Exercised.1	F.1	LowFat.Exercised.1	4.54	17.2	25	0.436	1145.76	19283	Evans.1.22
Evans.1.3	CATCGCGTTGAC	CCGGACTACHVGGGTWTCTAAT	3	13	HighFat	Sedentary	A	1	HighFat.Sedentary	HighFat.A	HighFat.1	Sedentary.A	Sedentary.1	A.1	HighFat.Sedentary.1	4.212	18.9	35	1.708	NA	29820	Evans.1.3
Evans.1.7	CCTAGTAAGCTG	CCGGACTACHVGGGTWTCTAAT	7	14	HighFat	Sedentary	B	1	HighFat.Sedentary	HighFat.B	HighFat.1	Sedentary.B	Sedentary.1	B.1	HighFat.Sedentary.1	3.671	18.3	28.7	0.913	NA	33248	Evans.1.7
Evans.1.11	TTACCTTACACC	CCGGACTACHVGGGTWTCTAAT	11	15	HighFat	Sedentary	C	1	HighFat.Sedentary	HighFat.C	HighFat.1	Sedentary.C	Sedentary.1	C.1	HighFat.Sedentary.1	4.417	16.6	31	1.291	NA	27038	Evans.1.11
Evans.1.15	CACGTGACATGT	CCGGACTACHVGGGTWTCTAAT	15	16	HighFat	Sedentary	D	1	HighFat.Sedentary	HighFat.D	HighFat.1	Sedentary.D	Sedentary.1	D.1	HighFat.Sedentary.1	4.095	17.9	36.7	2.608	NA	36855	Evans.1.15
Evans.1.19	ACCGGAGTAGGA	CCGGACTACHVGGGTWTCTAAT	19	17	HighFat	Sedentary	E	1	HighFat.Sedentary	HighFat.E	HighFat.1	Sedentary.E	Sedentary.1	E.1	HighFat.Sedentary.1	4.728	17	32.7	1.413	NA	34628	Evans.1.19
Evans.1.23	TGACCGGCTGTT	CCGGACTACHVGGGTWTCTAAT	23	18	HighFat	Sedentary	F	1	HighFat.Sedentary	HighFat.F	HighFat.1	Sedentary.F	Sedentary.1	F.1	HighFat.Sedentary.1	4.205	18.7	34.3	2.436	NA	33615	Evans.1.23
Evans.1.4	GCACATAGTCGT	CCGGACTACHVGGGTWTCTAAT	4	19	HighFat	Exercised	A	1	HighFat.Exercised	HighFat.A	HighFat.1	Exercised.A	Exercised.1	A.1	HighFat.Exercised.1	4.526	17.6	24.4	0.505	685.6	21825	Evans.1.4
Evans.1.8	TTACCGACGAGT	CCGGACTACHVGGGTWTCTAAT	8	20	HighFat	Exercised	B	1	HighFat.Exercised	HighFat.B	HighFat.1	Exercised.B	Exercised.1	B.1	HighFat.Exercised.1	4.269	18.5	27.4	0.471	2482	25043	Evans.1.8
Evans.1.12	TGACTAATGGCC	CCGGACTACHVGGGTWTCTAAT	12	21	HighFat	Exercised	C	1	HighFat.Exercised	HighFat.C	HighFat.1	Exercised.C	Exercised.1	C.1	HighFat.Exercised.1	4.896	17.6	26	0.599	1221.6	16200	Evans.1.12
Evans.1.16	CACAGTTGAAGT	CCGGACTACHVGGGTWTCTAAT	16	22	HighFat	Exercised	D	1	HighFat.Exercised	HighFat.D	HighFat.1	Exercised.D	Exercised.1	D.1	HighFat.Exercised.1	3.074	18.5	33.7	1.906	671.4	34845	Evans.1.16
Evans.1.20	TGAGGACTACCT	CCGGACTACHVGGGTWTCTAAT	20	23	HighFat	Exercised	E	1	HighFat.Exercised	HighFat.E	HighFat.1	Exercised.E	Exercised.1	E.1	HighFat.Exercised.1	4.643	16.8	27.2	0.704	2118.39	22433	Evans.1.20
Evans.1.24	GGAGGAGCAATA	CCGGACTACHVGGGTWTCTAAT	24	24	HighFat	Exercised	F	1	HighFat.Exercised	HighFat.F	HighFat.1	Exercised.F	Exercised.1	F.1	HighFat.Exercised.1	5.152	18.1	28.6	0.918	1197.7	24525	Evans.1.24
Evans.7.1	AGCGACGAAGAC	CCGGACTACHVGGGTWTCTAAT	1	25	LowFat	Sedentary	A	7	LowFat.Sedentary	LowFat.A	LowFat.7	Sedentary.A	Sedentary.7	A.7	LowFat.Sedentary.7	4.588	18.2	28.5	0.925	NA	22328	Evans.7.1
Evans.7.5	TTGGATTGAACG	CCGGACTACHVGGGTWTCTAAT	5	26	LowFat	Sedentary	B	7	LowFat.Sedentary	LowFat.B	LowFat.7	Sedentary.B	Sedentary.7	B.7	LowFat.Sedentary.7	4.6	19.1	28.2	0.744	NA	20400	Evans.7.5
Evans.7.9	TACAGTTACGCG	CCGGACTACHVGGGTWTCTAAT	9	27	LowFat	Sedentary	C	7	LowFat.Sedentary	LowFat.C	LowFat.7	Sedentary.C	Sedentary.7	C.7	LowFat.Sedentary.7	5.085	16.1	25.2	0.576	NA	22575	Evans.7.9
Evans.7.13	CTTCGACTTTCC	CCGGACTACHVGGGTWTCTAAT	13	28	LowFat	Sedentary	D	7	LowFat.Sedentary	LowFat.D	LowFat.7	Sedentary.D	Sedentary.7	D.7	LowFat.Sedentary.7	4.5	18.7	27.6	0.824	NA	25898	Evans.7.13

Evans.7.17	CCTCTGAGAGCT	CCGGACTACHVGGGTWTCTAAT	17	29	LowFat	Sedentary	E	7	LowFat.Sedentary	LowFat.E	LowFat.7	Sedentary.E	Sedentary.7	E.7	LowFat.Sedentary.7	4.38	17.7	26.2	0.489	NA	18818	Evans.7.17
Evans.7.21	CGGCCTAAGTTC	CCGGACTACHVGGGTWTCTAAT	21	30	LowFat	Sedentary	F	7	LowFat.Sedentary	LowFat.F	LowFat.7	Sedentary.F	Sedentary.7	F.7	LowFat.Sedentary.7	4.639	18.5	26.9	0.486	NA	17880	Evans.7.21
Evans.7.2	CTTCCCTAACTC	CCGGACTACHVGGGTWTCTAAT	2	31	LowFat	Exercised	A	7	LowFat.Exercised	LowFat.A	LowFat.7	Exercised.A	Exercised.7	A.7	LowFat.Exercised.7	4.644	17.9	26	NA	1046	19313	Evans.7.2
Evans.7.6	GATATACCAGTG	CCGGACTACHVGGGTWTCTAAT	6	32	LowFat	Exercised	B	7	LowFat.Exercised	LowFat.B	LowFat.7	Exercised.B	Exercised.7	B.7	LowFat.Exercised.7	4.347	19	24.9	0.485	1056	18188	Evans.7.6
Evans.7.10	CAAGCCCTAGTA	CCGGACTACHVGGGTWTCTAAT	10	33	LowFat	Exercised	C	7	LowFat.Exercised	LowFat.C	LowFat.7	Exercised.C	Exercised.7	C.7	LowFat.Exercised.7	4.973	18.4	25.8	0.333	976.7	29040	Evans.7.10
Evans.7.14	GTCATAAGAACC	CCGGACTACHVGGGTWTCTAAT	14	34	LowFat	Exercised	D	7	LowFat.Exercised	LowFat.D	LowFat.7	Exercised.D	Exercised.7	D.7	LowFat.Exercised.7	4.031	18.2	24.7	0.578	1406.8	18900	Evans.7.14
Evans.7.18	CCTCGATGCAGT	CCGGACTACHVGGGTWTCTAAT	18	35	LowFat	Exercised	E	7	LowFat.Exercised	LowFat.E	LowFat.7	Exercised.E	Exercised.7	E.7	LowFat.Exercised.7	4.543	19	25.6	0.539	1119.899	23865	Evans.7.18
Evans.7.22	AGCGCTCACATC	CCGGACTACHVGGGTWTCTAAT	22	36	LowFat	Exercised	F	7	LowFat.Exercised	LowFat.F	LowFat.7	Exercised.F	Exercised.7	F.7	LowFat.Exercised.7	4.808	17.2	25	0.436	1145.76	19283	Evans.7.22
Evans.7.3	TGGAAGAACGGC	CCGGACTACHVGGGTWTCTAAT	3	37	HighFat	Sedentary	A	7	HighFat.Sedentary	HighFat.A	HighFat.7	Sedentary.A	Sedentary.7	A.7	HighFat.Sedentary.7	5.294	18.9	35	1.708	NA	29820	Evans.7.3
Evans.7.7	AACAAACTGCCA	CCGGACTACHVGGGTWTCTAAT	7	38	HighFat	Sedentary	B	7	HighFat.Sedentary	HighFat.B	HighFat.7	Sedentary.B	Sedentary.7	B.7	HighFat.Sedentary.7	4.906	18.3	28.7	0.913	NA	33248	Evans.7.7
Evans.7.11	CTGAGCTCTGCA	CCGGACTACHVGGGTWTCTAAT	11	39	HighFat	Sedentary	C	7	HighFat.Sedentary	HighFat.C	HighFat.7	Sedentary.C	Sedentary.7	C.7	HighFat.Sedentary.7	5.932	16.6	31	1.291	NA	27038	Evans.7.11
Evans.7.15	GTCCGCAAGTTA	CCGGACTACHVGGGTWTCTAAT	15	40	HighFat	Sedentary	D	7	HighFat.Sedentary	HighFat.D	HighFat.7	Sedentary.D	Sedentary.7	D.7	HighFat.Sedentary.7	4.877	17.9	36.7	2.608	NA	36855	Evans.7.15
Evans.7.19	GCGGACTATTCA	CCGGACTACHVGGGTWTCTAAT	19	41	HighFat	Sedentary	E	7	HighFat.Sedentary	HighFat.E	HighFat.7	Sedentary.E	Sedentary.7	E.7	HighFat.Sedentary.7	4.646	17	32.7	1.413	NA	34628	Evans.7.19
Evans.7.23	TGGTTATGGCAC	CCGGACTACHVGGGTWTCTAAT	23	42	HighFat	Sedentary	F	7	HighFat.Sedentary	HighFat.F	HighFat.7	Sedentary.F	Sedentary.7	F.7	HighFat.Sedentary.7	5.931	18.7	34.3	2.436	NA	33615	Evans.7.23
Evans.7.4	GCTAGACACTAC	CCGGACTACHVGGGTWTCTAAT	4	43	HighFat	Exercised	A	7	HighFat.Exercised	HighFat.A	HighFat.7	Exercised.A	Exercised.7	A.7	HighFat.Exercised.7	5.556	17.6	24.4	0.505	685.6	21825	Evans.7.4
Evans.7.8	GTAGACATGTGT	CCGGACTACHVGGGTWTCTAAT	8	44	HighFat	Exercised	B	7	HighFat.Exercised	HighFat.B	HighFat.7	Exercised.B	Exercised.7	B.7	HighFat.Exercised.7	5.403	18.5	27.4	0.471	2482	25043	Evans.7.8
Evans.7.12	TAGTGTCGGATC	CCGGACTACHVGGGTWTCTAAT	12	45	HighFat	Exercised	C	7	HighFat.Exercised	HighFat.C	HighFat.7	Exercised.C	Exercised.7	C.7	HighFat.Exercised.7	5.074	17.6	26	0.599	1221.6	16200	Evans.7.12
Evans.7.16	CGTAGAGCTCTC	CCGGACTACHVGGGTWTCTAAT	16	46	HighFat	Exercised	D	7	HighFat.Exercised	HighFat.D	HighFat.7	Exercised.D	Exercised.7	D.7	HighFat.Exercised.7	5.029	18.5	33.7	1.906	671.4	34845	Evans.7.16
Evans.7.20	CGTGCACAATTG	CCGGACTACHVGGGTWTCTAAT	20	47	HighFat	Exercised	E	7	HighFat.Exercised	HighFat.E	HighFat.7	Exercised.E	Exercised.7	E.7	HighFat.Exercised.7	5.735	16.8	27.2	0.704	2118.39	22433	Evans.7.20
Evans.7.24	CGAGGTTCTGAT	CCGGACTACHVGGGTWTCTAAT	24	48	HighFat	Exercised	F	7	HighFat.Exercised	HighFat.F	HighFat.7	Exercised.F	Exercised.7	F.7	HighFat.Exercised.7	5.289	18.1	28.6	0.918	1197.7	24525	Evans.7.24
Evans.12.1	AACTCCTGTGGA	CCGGACTACHVGGGTWTCTAAT	1	49	LowFat	Sedentary	A	12	LowFat.Sedentary	LowFat.A	LowFat.12	Sedentary.A	Sedentary.12	A.12	LowFat.Sedentary.12	4.306	18.2	28.5	0.925	NA	22328	Evans.12.1
Evans.12.5	ATGGCCTGACTA	CCGGACTACHVGGGTWTCTAAT	5	50	LowFat	Sedentary	B	12	LowFat.Sedentary	LowFat.B	LowFat.12	Sedentary.B	Sedentary.12	B.12	LowFat.Sedentary.12	3.995	19.1	28.2	0.744	NA	20400	Evans.12.5
Evans.12.9	TAGCGCGAACTT	CCGGACTACHVGGGTWTCTAAT	9	51	LowFat	Sedentary	C	12	LowFat.Sedentary	LowFat.C	LowFat.12	Sedentary.C	Sedentary.12	C.12	LowFat.Sedentary.12	4.45	16.1	25.2	0.576	NA	22575	Evans.12.9
Evans.12.13	CCACCCAGTAAC	CCGGACTACHVGGGTWTCTAAT	13	52	LowFat	Sedentary	D	12	LowFat.Sedentary	LowFat.D	LowFat.12	Sedentary.D	Sedentary.12	D.12	LowFat.Sedentary.12	4.128	18.7	27.6	0.824	NA	25898	Evans.12.13
Evans.12.17	AGCAGGCACGAA	CCGGACTACHVGGGTWTCTAAT	17	53	LowFat	Sedentary	E	12	LowFat.Sedentary	LowFat.E	LowFat.12	Sedentary.E	Sedentary.12	E.12	LowFat.Sedentary.12	3.886	17.7	26.2	0.489	NA	18818	Evans.12.17
Evans.12.21	TCGAGCCGATCT	CCGGACTACHVGGGTWTCTAAT	21	54	LowFat	Sedentary	F	12	LowFat.Sedentary	LowFat.F	LowFat.12	Sedentary.F	Sedentary.12	F.12	LowFat.Sedentary.12	4.037	18.5	26.9	0.486	NA	17880	Evans.12.21
Evans.12.2	TAATGGTCGTAG	CCGGACTACHVGGGTWTCTAAT	2	55	LowFat	Exercised	A	12	LowFat.Exercised	LowFat.A	LowFat.12	Exercised.A	Exercised.12	A.12	LowFat.Exercised.12	4.428	17.9	26	NA	1046	19313	Evans.12.2
Evans.12.6	ACGCACATACAA	CCGGACTACHVGGGTWTCTAAT	6	56	LowFat	Exercised	B	12	LowFat.Exercised	LowFat.B	LowFat.12	Exercised.B	Exercised.12	B.12	LowFat.Exercised.12	3.817	19	24.9	0.485	1056	18188	Evans.12.6
Evans.12.10	CATACACGCACC	CCGGACTACHVGGGTWTCTAAT	10	57	LowFat	Exercised	C	12	LowFat.Exercised	LowFat.C	LowFat.12	Exercised.C	Exercised.12	C.12	LowFat.Exercised.12	3.805	18.4	25.8	0.333	976.7	29040	Evans.12.10
Evans.12.14	ATATCGCGATGA	CCGGACTACHVGGGTWTCTAAT	14	58	LowFat	Exercised	D	12	LowFat.Exercised	LowFat.D	LowFat.12	Exercised.D	Exercised.12	D.12	LowFat.Exercised.12	4.482	18.2	24.7	0.578	1406.8	18900	Evans.12.14
Evans.12.18	TACGCAGCACTA	CCGGACTACHVGGGTWTCTAAT	18	59	LowFat	Exercised	E	12	LowFat.Exercised	LowFat.E	LowFat.12	Exercised.E	Exercised.12	E.12	LowFat.Exercised.12	4.63	19	25.6	0.539	1119.899	23865	Evans.12.18

Evans.12.22	CTCATCATGTTC	CCGGACTACHVGGGTWTCTAAT	22	60	LowFat	Exercised	F	12	LowFat.Exercised	LowFat.F	LowFat.12	Exercised.F	Exercised.12	F.12	LowFat.Exercised.12	3.894	17.2	25	0.436	1145.76	19283	Evans.12.22
Evans.12.3	TTGCACCGTCGA	CCGGACTACHVGGGTWTCTAAT	3	61	HighFat	Sedentary	A	12	HighFat.Sedentary	HighFat.A	HighFat.12	Sedentary.A	Sedentary.12	A.12	HighFat.Sedentary.12	3.73	18.9	35	1.708	NA	29820	Evans.12.3
Evans.12.7	TGAGTGGTCTGT	CCGGACTACHVGGGTWTCTAAT	7	62	HighFat	Sedentary	B	12	HighFat.Sedentary	HighFat.B	HighFat.12	Sedentary.B	Sedentary.12	B.12	HighFat.Sedentary.12	4.649	18.3	28.7	0.913	NA	33248	Evans.12.7
Evans.12.11	ACCTCAGTCAAG	CCGGACTACHVGGGTWTCTAAT	11	63	HighFat	Sedentary	C	12	HighFat.Sedentary	HighFat.C	HighFat.12	Sedentary.C	Sedentary.12	C.12	HighFat.Sedentary.12	4.845	16.6	31	1.291	NA	27038	Evans.12.11
Evans.12.15	CGCCGGTAATCT	CCGGACTACHVGGGTWTCTAAT	15	64	HighFat	Sedentary	D	12	HighFat.Sedentary	HighFat.D	HighFat.12	Sedentary.D	Sedentary.12	D.12	HighFat.Sedentary.12	4.936	17.9	36.7	2.608	NA	36855	Evans.12.15
Evans.12.19	CGCTTAGTGCTG	CCGGACTACHVGGGTWTCTAAT	19	65	HighFat	Sedentary	E	12	HighFat.Sedentary	HighFat.E	HighFat.12	Sedentary.E	Sedentary.12	E.12	HighFat.Sedentary.12	5.006	17	32.7	1.413	NA	34628	Evans.12.19
Evans.12.23	CCAGGGACTTCT	CCGGACTACHVGGGTWTCTAAT	23	66	HighFat	Sedentary	F	12	HighFat.Sedentary	HighFat.F	HighFat.12	Sedentary.F	Sedentary.12	F.12	HighFat.Sedentary.12	4.735	18.7	34.3	2.436	NA	33615	Evans.12.23
Evans.12.4	TGCTACAGACGT	CCGGACTACHVGGGTWTCTAAT	4	67	HighFat	Exercised	A	12	HighFat.Exercised	HighFat.A	HighFat.12	Exercised.A	Exercised.12	A.12	HighFat.Exercised.12	5.327	17.6	24.4	0.505	685.6	21825	Evans.12.4
Evans.12.8	GATAGCACTCGT	CCGGACTACHVGGGTWTCTAAT	8	68	HighFat	Exercised	B	12	HighFat.Exercised	HighFat.B	HighFat.12	Exercised.B	Exercised.12	B.12	HighFat.Exercised.12	5.749	18.5	27.4	0.471	2482	25043	Evans.12.8
Evans.12.12	TCGACCAAACAC	CCGGACTACHVGGGTWTCTAAT	12	69	HighFat	Exercised	C	12	HighFat.Exercised	HighFat.C	HighFat.12	Exercised.C	Exercised.12	C.12	HighFat.Exercised.12	5.424	17.6	26	0.599	1221.6	16200	Evans.12.12
Evans.12.16	CCGATGCCTTGA	CCGGACTACHVGGGTWTCTAAT	16	70	HighFat	Exercised	D	12	HighFat.Exercised	HighFat.D	HighFat.12	Exercised.D	Exercised.12	D.12	HighFat.Exercised.12	5.158	18.5	33.7	1.906	671.4	34845	Evans.12.16
Evans.12.20	CAAAGTTTGCGA	CCGGACTACHVGGGTWTCTAAT	20	71	HighFat	Exercised	E	12	HighFat.Exercised	HighFat.E	HighFat.12	Exercised.E	Exercised.12	E.12	HighFat.Exercised.12	5.253	16.8	27.2	0.704	2118.39	22433	Evans.12.20
Evans.12.24	GCAATCCTTGCG	CCGGACTACHVGGGTWTCTAAT	24	72	HighFat	Exercised	F	12	HighFat.Exercised	HighFat.F	HighFat.12	Exercised.F	Exercised.12	F.12	HighFat.Exercised.12	5.314	18.1	28.6	0.918	1197.7	24525	Evans.12.24

```
mkdir 00_rawseqs
cd 00_rawseqs
wget ftp://ftp.igsb.anl.gov/jobs/cc8b5e8e68cedf34099c4063de229bd4/Evans_16S/Undetermined_S0_L001_*.fastq.gz
cd ..

mkdir scripts
wget -q -O scripts/fastq-join https://www.dropbox.com/s/m51lmz4vu9ooc2o/fastq-join?dl=1
wget -q -O scripts/fastq-barcode.pl https://www.dropbox.com/s/hk33ovypzmev938/fastq-barcode.pl?dl=1
wget -q -O scripts/fastq_split.pl https://www.dropbox.com/s/pkqw7sh40nh02q9/fastq_split.pl?dl=1
wget -q -O scripts/ttest_otu_table.pl https://www.dropbox.com/s/tpv8ptppyf03in3/ttest_otu_table.pl?dl=1
chmod a+x scripts/*

mkdir 01_joined
gunzip -c 00_rawseqs/*_L001_I1* > 01_joined/barcodes.fastq
gunzip -c 00_rawseqs/*_L001_R1* > 01_joined/read1.fastq
gunzip -c 00_rawseqs/*_L001_R2* > 01_joined/read2.fastq
./scripts/fastq-join 01_joined/read1.fastq 01_joined/read2.fastq -o 01_joined/out.%.fastq > 01_joined/out.stats.txt
./scripts/fastq-barcode.pl 01_joined/barcodes.fastq 01_joined/out.join.fastq > 01_joined/out.barcodes.fastq

mkdir 02_demult
./scripts/fastq_split.pl -m MappingFile.txt -i 01_joined/out.join.fastq -b 01_joined/out.barcodes.fastq -o 02_demult/seqs.fna

pick_otus.py -i 02_demult/seqs.fna -o 03_otus
perl -nae 'print $_ if (@F - 1 >= 2);' < 03_otus/seqs_otus.txt > 03_otus/filtered_seqs_otus.txt

mkdir 04_rep_set
pick_rep_set.py -i 03_otus/filtered_seqs_otus.txt -f 02_demult/seqs.fna -l 04_rep_set/rep_set.log -o 04_rep_set/rep_set.fasta
assign_taxonomy.py -i 04_rep_set/rep_set.fasta -o 05_taxonomy -t /usr/local/bin/qiime-1.5.0/gg_otus-16oct2012-release/taxonomy/97_rdp_train.txt -r
/usr/local/bin/qiime-1.5.0/gg_otus-16oct2012-release/rep_set/97_otus.fasta

mkdir 06_otu_table
make_otu_table.py -i 03_otus/filtered_seqs_otus.txt -t 05_taxonomy/rep_set_tax_assignments.txt -o 06_otu_table/otu_table.biom

# Call "per_library_stats.py -i 06_otu_table/otu_table.biom" to see what depth to subsample to
single_rarefaction.py -i 06_otu_table/otu_table.biom -o 06_otu_table/otu_table_even.biom -d 1600
sort_otu_table.py -i 06_otu_table/otu_table_even.biom -o 06_otu_table/otu_table_even_sorted.biom -m MappingFile.txt -s Sort
mv 06_otu_table/otu_table_even_sorted.biom 06_otu_table/otu_table_even.biom

align_seqs.py -i 04_rep_set/rep_set.fasta -o 07_aligned_seqs -t /usr/local/bin/qiime-1.5.0/core_set_aligned.fasta.imputed
filter_alignment.py -i 07_aligned_seqs/rep_set_aligned.fasta -o 07_aligned_seqs

mkdir 08_phylogeny
make_phylogeny.py -i 07_aligned_seqs/rep_set_aligned_pfiltered.fasta -o 08_phylogeny/rep_set.tre

beta_diversity_through_plots.py -i 06_otu_table/otu_table_even.biom -t 08_phylogeny/rep_set.tre -m MappingFile.txt -o 09_beta_diversity -a -O 6
make_otu_network.py -m MappingFile.txt -i 06_otu_table/otu_table_even.biom -o 10_otu_network
summarize_taxa_through_plots.py -i 06_otu_table/otu_table_even.biom -o 11_taxa_summary -m MappingFile.txt

echo "alpha_diversity:metrics shannon,PD_whole_tree,chao1,observed_species" > alpha_params.txt
alpha_rarefaction.py -i 06_otu_table/otu_table.biom -m MappingFile.txt -o 12_alpha_rarefaction -p alpha_params.txt -t 08_phylogeny/rep_set.tre

jackknifed_beta_diversity.py -i 06_otu_table/otu_table_even.biom -t 08_phylogeny/rep_set.tre -m MappingFile.txt -o 13_jackknifed_bdiv -e 1000
make_bootstrapped_tree.py -m 13_jackknifed_bdiv/unweighted_unifrac/upgma_cmp/master_tree.tre -s
13_jackknifed_bdiv/unweighted_unifrac/upgma_cmp/jackknife_support.txt -o 13_jackknifed_bdiv/unweighted_unifrac/upgma_cmp/jackknife_named_nodes.pdf
make_bootstrapped_tree.py -m 13_jackknifed_bdiv/weighted_unifrac/upgma_cmp/master_tree.tre -s
13_jackknifed_bdiv/weighted_unifrac/upgma_cmp/jackknife_support.txt -o 13_jackknifed_bdiv/weighted_unifrac/upgma_cmp/jackknife_named_nodes.pdf

./scripts/ttest_otu_table.pl -i 06_otu_table/otu_table_even.biom -m MappingFile.txt -o 18_ttest_otu_table -r 1600 -p Mouse -x "NA" -t 80 -n

# Discrete Variables:
mkdir 15_boxplots
mkdir 16_otu_cat_sig
```

```
mkdir 17_compare_cat
for f in Mouse Diet Lifestyle Litter Week Diet.LifeStyle Diet.Litter Diet.Week LifeStyle.Litter LifeStyle.Week Litter.Week Diet.LifeStyle.Week
do
	make_distance_boxplots.py    -d 09_beta_diversity/unweighted_unifrac_dm.txt -m MappingFile.txt -o 15_boxplots --height 12 -f $f --sort

	otu_category_significance.py -i 06_otu_table/otu_table_even.biom            -m MappingFile.txt -s       ANOVA    -o
16_otu_cat_sig/anova_All.By.$f.txt -c $f
	otu_category_significance.py -i 06_otu_table/otu_table_even.biom            -m MappingFile.txt -s       g_test   -o
16_otu_cat_sig/gtest_All.By.$f.txt -c $f
	compare_categories.py        -i 09_beta_diversity/unweighted_unifrac_dm.txt -m MappingFile.txt --method adonis   -o 17_compare_cat/adonis_All.By.$f
-c $f
	compare_categories.py        -i 09_beta_diversity/unweighted_unifrac_dm.txt -m MappingFile.txt --method anosim   -o 17_compare_cat/anosim_All.By.$f
-c $f
	compare_categories.py        -i 09_beta_diversity/unweighted_unifrac_dm.txt -m MappingFile.txt --method mrpp     -o 17_compare_cat/mrpp_All.By.$f
-c $f
	compare_categories.py        -i 09_beta_diversity/unweighted_unifrac_dm.txt -m MappingFile.txt --method permdisp -o
17_compare_cat/permdisp_All.By.$f  -c $f
	compare_categories.py        -i 09_beta_diversity/unweighted_unifrac_dm.txt -m MappingFile.txt --method rda      -o 17_compare_cat/rda_All.By.$f
-c $f
done




mkdir 14_subgroups
# Mouse Diet Lifestyle Litter Week Diet.LifeStyle Diet.Litter Diet.Week LifeStyle.Litter LifeStyle.Week Litter.Week Diet.LifeStyle.Week

# Subdivide on Diet
export v='Diet'
mkdir 14_subgroups/$v
mkdir 14_subgroups/$v/Stats
mkdir 14_subgroups/$v/TTests
for c in LowFat HighFat
do
	filter_samples_from_otu_table.py -i 06_otu_table/otu_table_even.biom -o 14_subgroups/$v/$c.biom -m MappingFile.txt --output_mapping_fp
14_subgroups/$v/$c.map -s "$v:$c"
	sort_otu_table.py -i 14_subgroups/$v/$c.biom -o 14_subgroups/$v/$c.biom.sorted -m 14_subgroups/$v/$c.map -s Week
	mv 14_subgroups/$v/$c.biom.sorted 14_subgroups/$v/$c.biom
	beta_diversity_through_plots.py -i 14_subgroups/$v/$c.biom -t 08_phylogeny/rep_set.tre -m 14_subgroups/$v/$c.map -o 14_subgroups/$v/BetaDiversity_$c
--suppress_3d_plots -a -O 6
	summarize_taxa_through_plots.py -i 14_subgroups/$v/$c.biom -m 14_subgroups/$v/$c.map -o 14_subgroups/$v/TaxaSummaryBarPlots_$c

	./scripts/ttest_otu_table.pl -i 14_subgroups/$v/$c.biom -m 14_subgroups/$v/$c.map -o 14_subgroups/$v/TTests/$c -r 1600 -p Mouse -x "NA" -t 80 -n

	for f in Mouse Lifestyle Litter Week LifeStyle.Litter LifeStyle.Week Litter.Week
	do
		make_distance_boxplots.py -d 14_subgroups/$v/BetaDiversity_$c/unweighted_unifrac_dm.txt -m 14_subgroups/$v/$c.map -o
14_subgroups/$v/BoxPlots_$c --height 12 -f $f --sort

		otu_category_significance.py -i 14_subgroups/$v/$c.biom                                    -m 14_subgroups/$v/$c.map -s       ANOVA    -o
14_subgroups/$v/Stats/anova_$c.By.$f.txt -c $f
		otu_category_significance.py -i 14_subgroups/$v/$c.biom                                    -m 14_subgroups/$v/$c.map -s       g_test   -o
14_subgroups/$v/Stats/gtest_$c.By.$f.txt -c $f
		compare_categories.py        -i 14_subgroups/$v/BetaDiversity_$c/unweighted_unifrac_dm.txt -m 14_subgroups/$v/$c.map --method adonis   -o
14_subgroups/$v/Stats/adonis_$c.By.$f    -c $f
		compare_categories.py        -i 14_subgroups/$v/BetaDiversity_$c/unweighted_unifrac_dm.txt -m 14_subgroups/$v/$c.map --method anosim   -o
14_subgroups/$v/Stats/anosim_$c.By.$f    -c $f
		compare_categories.py        -i 14_subgroups/$v/BetaDiversity_$c/unweighted_unifrac_dm.txt -m 14_subgroups/$v/$c.map --method mrpp     -o
14_subgroups/$v/Stats/mrpp_$c.By.$f      -c $f
		compare_categories.py        -i 14_subgroups/$v/BetaDiversity_$c/unweighted_unifrac_dm.txt -m 14_subgroups/$v/$c.map --method permdisp -o
14_subgroups/$v/Stats/permdisp_$c.By.$f  -c $f
```

```
		compare_categories.py 	-i 14_subgroups/$v/BetaDiversity_$c/unweighted_unifrac_dm.txt -m 14_subgroups/$v/$c.map --method rda 	-o 
14_subgroups/$v/Stats/rda_$c.By.$f 	-c $f
	done
done

# Subdivide on Lifestyle
export v='Lifestyle'
mkdir 14_subgroups/$v
mkdir 14_subgroups/$v/Stats
mkdir 14_subgroups/$v/TTests
for c in Sedentary Exercised
do
	filter_samples_from_otu_table.py -i 06_otu_table/otu_table_even.biom -o 14_subgroups/$v/$c.biom -m MappingFile.txt --output_mapping_fp 
14_subgroups/$v/$c.map -s "$v:$c"
	sort_otu_table.py -i 14_subgroups/$v/$c.biom -o 14_subgroups/$v/$c.biom.sorted -m 14_subgroups/$v/$c.map -s Week
	mv 14_subgroups/$v/$c.biom.sorted 14_subgroups/$v/$c.biom
	beta_diversity_through_plots.py -i 14_subgroups/$v/$c.biom -t 08_phylogeny/rep_set.tre -m 14_subgroups/$v/$c.map -o 14_subgroups/$v/BetaDiversity_$c 
--suppress_3d_plots -a -O 6
	summarize_taxa_through_plots.py -i 14_subgroups/$v/$c.biom -m 14_subgroups/$v/$c.map -o 14_subgroups/$v/TaxaSummaryBarPlots_$c

	./scripts/ttest_otu_table.pl -i 14_subgroups/$v/$c.biom -m 14_subgroups/$v/$c.map -o 14_subgroups/$v/TTests/$c -r 1600 -p Mouse -x "NA" -t 80 -n

	for f in Mouse Diet Litter Week Diet.Litter Diet.Week Litter.Week
	do
		make_distance_boxplots.py -d 14_subgroups/$v/BetaDiversity_$c/unweighted_unifrac_dm.txt -m 14_subgroups/$v/$c.map -o 
14_subgroups/$v/BoxPlots_$c --height 12 -f $f --sort

		otu_category_significance.py -i 14_subgroups/$v/$c.biom 				-m 14_subgroups/$v/$c.map -s 	ANOVA 	-o 
14_subgroups/$v/Stats/anova_$c.By.$f.txt -c $f
		otu_category_significance.py -i 14_subgroups/$v/$c.biom 				-m 14_subgroups/$v/$c.map -s 	g_test 	-o 
14_subgroups/$v/Stats/gtest_$c.By.$f.txt -c $f
		compare_categories.py 	-i 14_subgroups/$v/BetaDiversity_$c/unweighted_unifrac_dm.txt -m 14_subgroups/$v/$c.map --method adonis 	-o 
14_subgroups/$v/Stats/adonis_$c.By.$f 	-c $f
		compare_categories.py 	-i 14_subgroups/$v/BetaDiversity_$c/unweighted_unifrac_dm.txt -m 14_subgroups/$v/$c.map --method anosim 	-o 
14_subgroups/$v/Stats/anosim_$c.By.$f 	-c $f
		compare_categories.py 	-i 14_subgroups/$v/BetaDiversity_$c/unweighted_unifrac_dm.txt -m 14_subgroups/$v/$c.map --method mrpp 	-o 
14_subgroups/$v/Stats/mrpp_$c.By.$f 	-c $f
		compare_categories.py 	-i 14_subgroups/$v/BetaDiversity_$c/unweighted_unifrac_dm.txt -m 14_subgroups/$v/$c.map --method permdisp -o 
14_subgroups/$v/Stats/permdisp_$c.By.$f 	-c $f
		compare_categories.py 	-i 14_subgroups/$v/BetaDiversity_$c/unweighted_unifrac_dm.txt -m 14_subgroups/$v/$c.map --method rda 	-o 
14_subgroups/$v/Stats/rda_$c.By.$f 	-c $f
	done
done

# Subdivide on Week
export v='Week'
mkdir 14_subgroups/$v
mkdir 14_subgroups/$v/Stats
mkdir 14_subgroups/$v/TTests
for c in 1 7 12
do
	filter_samples_from_otu_table.py -i 06_otu_table/otu_table_even.biom -o 14_subgroups/$v/$c.biom -m MappingFile.txt --output_mapping_fp 
14_subgroups/$v/$c.map -s "$v:$c"
	sort_otu_table.py -i 14_subgroups/$v/$c.biom -o 14_subgroups/$v/$c.biom.sorted -m 14_subgroups/$v/$c.map -s Week
	mv 14_subgroups/$v/$c.biom.sorted 14_subgroups/$v/$c.biom
	beta_diversity_through_plots.py -i 14_subgroups/$v/$c.biom -t 08_phylogeny/rep_set.tre -m 14_subgroups/$v/$c.map -o 14_subgroups/$v/BetaDiversity_$c 
--suppress_3d_plots -a -O 6
	summarize_taxa_through_plots.py -i 14_subgroups/$v/$c.biom -m 14_subgroups/$v/$c.map -o 14_subgroups/$v/TaxaSummaryBarPlots_$c

	./scripts/ttest_otu_table.pl -i 14_subgroups/$v/$c.biom -m 14_subgroups/$v/$c.map -o 14_subgroups/$v/TTests/$c -r 1600 -p Mouse -x "NA" -t 80 -n

	for f in Mouse Diet Lifestyle Litter Diet.LifeStyle Diet.Litter LifeStyle.Litter
```

```
        do
                make_distance_boxplots.py -d 14_subgroups/$v/BetaDiversity_$c/unweighted_unifrac_dm.txt -m 14_subgroups/$v/$c.map -o
14_subgroups/$v/BoxPlots_$c --height 12 -f $f --sort

                otu_category_significance.py -i 14_subgroups/$v/$c.biom                                    -m 14_subgroups/$v/$c.map -s       ANOVA    -o
14_subgroups/$v/Stats/anova_$c.By.$f.txt -c $f
                otu_category_significance.py -i 14_subgroups/$v/$c.biom                                    -m 14_subgroups/$v/$c.map -s       g_test   -o
14_subgroups/$v/Stats/gtest_$c.By.$f.txt -c $f
                compare_categories.py        -i 14_subgroups/$v/BetaDiversity_$c/unweighted_unifrac_dm.txt -m 14_subgroups/$v/$c.map --method adonis   -o
14_subgroups/$v/Stats/adonis_$c.By.$f    -c $f
                compare_categories.py        -i 14_subgroups/$v/BetaDiversity_$c/unweighted_unifrac_dm.txt -m 14_subgroups/$v/$c.map --method anosim   -o
14_subgroups/$v/Stats/anosim_$c.By.$f    -c $f
                compare_categories.py        -i 14_subgroups/$v/BetaDiversity_$c/unweighted_unifrac_dm.txt -m 14_subgroups/$v/$c.map --method mrpp     -o
14_subgroups/$v/Stats/mrpp_$c.By.$f      -c $f
                compare_categories.py        -i 14_subgroups/$v/BetaDiversity_$c/unweighted_unifrac_dm.txt -m 14_subgroups/$v/$c.map --method permdisp -o
14_subgroups/$v/Stats/permdisp_$c.By.$f  -c $f
                compare_categories.py        -i 14_subgroups/$v/BetaDiversity_$c/unweighted_unifrac_dm.txt -m 14_subgroups/$v/$c.map --method rda      -o
14_subgroups/$v/Stats/rda_$c.By.$f       -c $f
        done
done
```

This file contains the computer commands used in the analysis of the sequencing dataset for the QIIME 1.5.0 software suite.[1] The reads from samples were clustered at 97% sequence identity into operational taxonomic units (OTUs) then aligned to the October 12th, 2012 Greengenes bacterial reference tree.[2] Please see the Bioinformatics section of the Methods for details.

Bibliography:
1. Caporaso JG, Kuczynski J, Stombaugh J, Bittinger K, Bushman FD, et al. (2010) QIIME allows analysis of high-throughput community sequencing data. Nature Methods 7: 335-336.
2. DeSantis TZ, Hugenholtz P, Larsen N, Rojas M, Brodie EL, et al. (2006) Greengenes, a chimera-checked 16S rRNA gene database and workbench compatible with ARB. Applied and environmental microbiology 72: 5069-5072.
